# A Scalable and Sustainable Synthesis of Indirubin Frameworks Enabled by Deep Eutectic Solvents

**DOI:** 10.1002/cssc.202502114

**Published:** 2025-12-08

**Authors:** Biagio Delvecchio, Luciana Cicco, Andrea Nicola Paparella, Gaetano Di Salvo, Filippo Maria Perna, Vito Capriati

**Affiliations:** ^1^ Dipartimento di Farmacia–Scienze del Farmaco Università degli Studi di Bari Aldo Moro, Consorzio CINMPIS Bari Italy; ^2^ Dieffetti Cosmetici S.r.l. Minervino Murge Italy

**Keywords:** deep eutectic solvents, green chemistry, heterocycles, indirubin, indole alkaloids

## Abstract

Indirubin, the active component of the traditional Chinese remedy *Dang Gui Long Hui Wan*, exhibits broad therapeutic potential. However, its scalable and sustainable synthesis remains challenging when using conventional methods. We report a green and efficient synthetic protocol using deep eutectic solvents (DESs) as environmentally benign media. Indirubin was synthesized from isatin using NaBH_4_ in a choline chloride/urea DES at 70°C under air, achieving a 70% overall yield in 24 h without chromatographic purification. This approach, combined with an optimized work‐up, significantly reduces organic solvent use, improving both process safety and environmental sustainability. The protocol is robust and scalable, as demonstrated by a pilot‐scale preparation (386 g of isatin in 1.94 kg of DES), and grants access to a variety of indirubin derivatives, such as 5,5′‐difluoro, 5,5′‐dibromo, 5,5′‐dimethoxy, 5,5′‐dimethyl, 5‐bromoindirubin, 3′‐oxime, and *N*‐alkylated analogs, the latter being of particular interest as photoswitchable molecular platforms. A comprehensive CHEM21 metrics assessment reveals a 3.7‐fold reduction in E‐factor and improved effective mass yield (EM) and process mass intensity (PMI) values compared to conventional methanol‐based methods, underscoring the reduced environmental footprint of this approach. Overall, this strategy provides a greener, safer, and industrially viable route to pharmaceutically relevant indirubin scaffolds, fully aligned with sustainable chemistry principles.

## Graphical abstract

A sustainable protocol for the synthesis of indirubin in a choline chloride/urea deep eutectic solvent at 70°C under air affords the product in 70% yield without chromatography. The method is robust, scalable to pilot scale, and enables late‐stage functionalization, granting access to a wide range of indirubin derivatives. CHEM21 green metrics validate its markedly lower environmental impact compared to conventional routes.

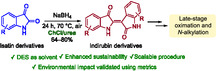

## 1 Introduction

**FIGURE 1:**
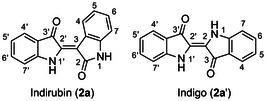
Chemical structures of the natural bis(indole) alkaloids: indirubin (**2a**) and indigo (**2a′**).

Indirubin (**2a**) and indigo (**2a′**) are representative members of the bis(indole) alkaloid family, formed as isomeric dimers of oxygenated indole units, linked by a carbon–carbon double bond at the 3–2′ position in **2a** and the 2–2′ position in **2a′** (Figure 1). Both compounds are key constituents of ancient dyes, most notably Indigo Blue, which for centuries has been extracted from the leaves of indigo‐producing plants such as *Isatis* spp. (Brassicaceae), *Polygonum* spp. (Polygonaceae), and *Indigofera* spp. (Fabaceae), and extensively used for fabric dyeing throughout history. In the pharmaceutical realm, **2a** is recognized as the active component of the traditional Chinese medicine *Dang Gui Long Hui Wan* [1], which is still prescribed today for the treatment of chronic myelocytic leukemia (CML). It functions as a selective and competitive inhibitor of the ATP‐binding sites of cyclin‐dependent kinases (CDKs), thereby effectively arresting the cell cycle at the late G1 and G2/M phases [2]. Animal studies have also demonstrated indirubin's anti‐inflammatory activity, linked to its ability to inhibit *γ*‐interferon production. Furthermore, in vitro studies have shown that indirubin can inhibit glycogen synthase kinase‐3*β* (GSK‐3*β*), a key regulator involved in the pathogenesis of Alzheimer's disease and diabetes through its role in cell cycle signaling pathways [3].

Beyond oncology, **2a** has demonstrated promising therapeutic potential in the treatment of psoriasis. Its multifaceted mechanism of action involves the regulation of keratinocyte (KC) proliferation and differentiation, inhibition of proinflammatory cytokine production, reduction of angiogenesis, modulation of immune responses, and restoration of epidermal barrier function [4, 5]. A clinical study involving 51 patients confirmed that a topical indigo naturalis ointment containing compound **2a** is a safe and effective therapy for plaque psoriasis, exhibiting minimal side effects [6].

Importantly, in recent years, indigoid dyes have also emerged as a particularly promising class of visible‐light‐responsive chromophores for photoswitching applications [7, 8, 9]. Unlike many conventional photoswitches that require ultraviolet activation, indigoid derivatives display intense coloration and undergo reversible isomerization under visible light, making them especially suitable for cutting‐edge applications in materials science and biological environments, where low‐energy activation is crucial.

**SCHEME 1:**
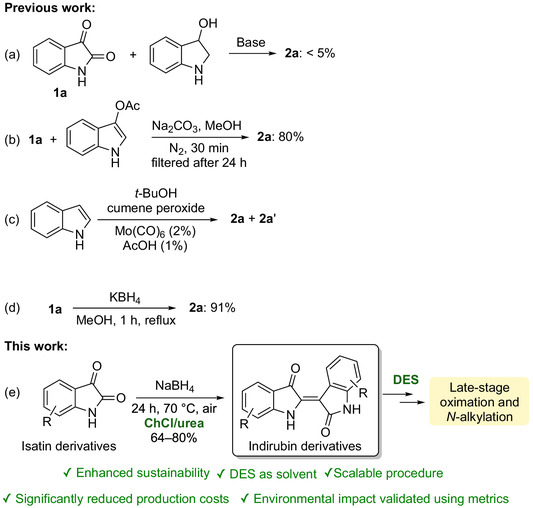
(a) Classical Baeyer–Emmerling synthesis of indirubin (**2a**). (b) Synthesis of **2a** via coupling of isatin (**1a**) with 3‐acetoxyindole in MeOH. (c) Formation of a mixture of **2a** and indigo (**2a′**) by reaction of indole with Mo(CO)_6_ and cumene hydroperoxide. (d) Synthesis of **2a** via reductive dimerization of **1a** with KBH_4_ in MeOH under reflux. (e) Synthesis of indirubin derivatives via reductive dimerization of isatin derivatives with NaBH_4_ in ChCl/urea at 70°C.

Currently, **2a** is obtained either through extraction from natural sources or as a minor byproduct in synthetic processes designed primarily for indigo production. However, extraction methods present several challenges, including dependence on seasonality, land availability, the need for fertilizers, and the use of high temperatures and volatile organic compounds (VOCs) [10]. Historically, the “Baeyer–Emmerling method” (1880)—a base‐catalyzed reaction between isatin (**1a**) and 3‐hydroxyindole, generated in situ under reducing conditions—has served as the foundational synthetic route (Scheme 1a) [11, 12]. Nonetheless, indirubin is generated only in very low yields (<5%), and the limited availability of indoxyl precursors remains a significant bottleneck. Alternative synthetic approaches have been developed, including the coupling of **1a** with 3‐acetoxyindole in MeOH using Na_2_CO_3_ as base (Scheme 1b) [13, 14], and the condensation of **1a** with 2‐oxindole in the presence of PCl_5_ [15]. More recently, oxidative strategies starting from indole have gained attention. One such method involves the reaction of indole with molybdenum hexacarbonyl [Mo(CO)_6_] and cumene hydroperoxide to afford **2a** and **2a′** (Scheme 1c). Careful control of the reaction temperature (40°C for 120 h) allows, at best, the formation of an equimolar mixture of **2a** and **2a′** [16, 17]. Additionally, **2a** can be obtained—albeit in low yield (~6%)—through the reaction of indole‐3‐carbaldehyde with oxone in acetonitrile [18].

Enzyme‐mediated strategies have also gained considerable interest, drawing inspiration from natural biosynthetic pathways, in which the hydrolysis of indican (indoxyl‐*β*‐D‐glucoside) leads to the formation of **2a** [19]. Plant tissue cultures supplemented with indole derivatives, as well as cytochrome P450‐catalyzed oxidations, have enabled the in situ production of **2a** alongside **2a′** [20, 21, 22]. Moreover, recent findings indicate that the presence of cysteine can influence the selectivity of flavin‐containing monooxygenases, favoring the formation of indirubin [23, 24]. Despite their biocatalytic sophistication, these approaches often yield **2a** only as a minor product and are limited by high enzyme costs and scalability issues.

In 2017, Liu et al. reported an interesting reductive dimerization of **1a**, affording **2a** in 91% yields by refluxing **1a** with KBH_4_ in MeOH (Scheme 1d) [25]. However, the use of MeOH remains a significant drawback from an environmental perspective. MeOH is a volatile, highly flammable liquid and vapor, as well as a neurotoxic solvent subject to strict regulations. Due to its potential for misuse and harmful effects, its use has been increasingly restricted in consumer products and certain industrial applications. Despite significant advances, existing methodologies continue to face major limitations, including the scarcity of starting materials, harsh reaction conditions, multistep procedures, low overall yields, and reliance on hazardous solvents and reagents. These challenges hinder not only synthetic efficiency but also the broader biological screening and development of new derivatives.

The widespread use of petroleum‐derived VOCs in organic synthesis significantly contributes to environmental pollution, contaminating water systems and exacerbating air quality issues. Given that solvents typically account for 80–90% of the mass input in chemical processes and are also important in influencing the kinetics and thermodynamics of chemical reactions, the development of “green” alternatives has become increasingly crucial [26, 27, 28, 29, 30]. Green solvents are typically defined by their low volatility and toxicity, nonflammability, renewable sourcing, and cost‐effectiveness [31, 32]. Among the most promising alternatives to VOCs are deep eutectic solvents (DESs), liquid mixtures usually obtained by combining a hydrogen bond donor with a hydrogen bond acceptor, resulting in a eutectic point significantly lower than that of an ideal liquid mixture of the individual components. When derived from naturally occurring compounds—such as polyols, amino alcohols, carboxylic acids, and urea derivatives—DESs present additional advantages, including biodegradability, low toxicity, and affordability, making them particularly attractive for sustainable chemistry applications [33, 34, 35]. Despite their remarkable potential, the application of DESs in the synthesis of active pharmaceutical ingredients (APIs) remains largely underexplored [36, 37, 38, 39, 40, 41, 42, 43, 44, 45]. Motivated by our long‐standing commitment to the sustainable synthesis of functionalized organic compounds, key building blocks, and pharmaceutically relevant molecules [46, 47, 48, 49, 50, 51, 52, 53, 54], and inspired by the notable pharmacological potential of indirubin derivatives, we set out to develop a novel, environmentally benign synthetic strategy for these compounds that addresses both efficiency and scalability demands.

Herein, we disclose the first sustainable protocol for the preparation and isolation of **2a** employing a choline chloride (ChCl)/urea DES as the reaction medium (Scheme 1e) [55]. This innovative approach (i) significantly reduces production costs, (ii) minimizes environmental impact, and (iii) obviates the need for chromatographic purification or extraction with VOCs, thereby markedly enhancing the sustainability profile compared to conventional methods. The reduced environmental impact of this newly developed approach was quantitatively validated using the CHEM21 Metrics Toolkit, with metrics applied at both first and second pass [56, 57]. The methodology has demonstrated remarkable robustness and versatility, enabling the reproducible synthesis of indirubin analogs bearing alkyl, halide, or methoxy substituents at the 5 and 5′ positions. Scale‐up studies up to the pilot scale—processing 386 g of starting material in 1.94 kg of DES—have further confirmed the practicality and potential of this approach for industrial applications. Notably, the use of DESs has also facilitated the efficient preparation of pharmacologically relevant derivatives, including 5‐bromoindirubin and indirubin‐3′‐oxime, as well as *N*‐monoalkylated and *N*,*N*′‐dialkylated indirubin analogs, which are currently under investigation for their potential as photoswitchable molecular platforms [7, 8, 9].

## 2 Results and Discussion

Inspired by the methodology developed by Liu [25], we initiated our investigation by evaluating the reduction of isatin (**1a**, 0.5 mmol) in various DESs using a hydride‐based reducing agent (500 mg of DES per reaction, Table 1). After 24 h of stirring, the reaction mixture was quenched with water and extracted with EtOAc. Following evaporation of the organic layer, the crude product was analyzed by ^1^H NMR spectroscopy to determine the yield of indirubin (**2a**). Among the eutectic mixtures tested, the combination of ChCl and urea in a 1:2 molar ratio proved to be the most effective. When 0.25 equiv of NaBH_4_ was used at 35°C, **2a** was obtained in 22% yield (Table 1, entry 1). In contrast, only trace amounts of **2a** were detected in the ChCl/glycerol (Gly) (1:2 mol/mol) system (Table 1, entry 2), and no product formation was observed in either choline acetate/urea (1:2 mol/mol) or ChCl/ethylene glycol (1:2 mol/mol) mixtures (Table 1, entries 3 and 4). Subsequent optimization studies focused on the ChCl/urea system. Increasing the amount of NaBH_4_ to 0.5 and 1.0 equiv led to improved yields of 27% and 38%, respectively (Table 1, entries 5 and 6). Under the same conditions, the use of ChCl/Gly and 1.0 equiv of NaBH_4_ resulted in a considerably lower yield (11%, Table 1, entry 7). Replacement of NaBH_4_ with KBH_4_ did not lead to significant changes in reactivity (Table 1, entry 8). Temperature was found to have a profound effect on reaction efficiency. Increasing the reaction temperature up to 70°C, under otherwise identical conditions, raised the yield of **2a** to 74% (Table 1, entries 9 and 10). However, further increasing the temperature or extending the reaction time up to 48 h did not provide any additional benefit (Table 1, entries 11 and 12). Likewise, increasing the amount of NaBH_4_ beyond 1.0 equivalent did not significantly improve the yield (Table 1, entry 13). Conversely, decreasing the amount of NaBH_4_ to 0.5 or 0.25 equivalents led to a marked drop in yield, affording **2a** in 40% and 10%, respectively (Table 1, entries 14,15). The use of NaBH_3_CN as an alternative reducing agent proved ineffective, affording **2a** in less than 5% yield (Table 1, entry 16). The optimized reaction conditions were further validated by scaling up the transformation: starting from 1.0 g of **1a** in 5.0 g of ChCl/urea (1:2 mol/mol), the reaction furnished **2a** in comparable yield (70%) (vide infra) to that obtained on a smaller scale, confirming the practicality and robustness of the method (Table 1, entry 17).

**TABLE 1:** Optimization of reaction conditions.^a^

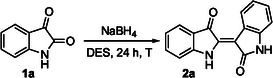				
Entry	DES	NaBH_4_ (equiv)	T (°C)	**2a** yield (%)^b^
1	ChCl/urea	0.25	35	22
2	ChCl/Gly	0.25	35	5
3	ChOAc/urea	0.25	35	NR^c^
4	ChCl/EG	0.25	35	NR^c^
5	ChCl/urea	0.5	35	27
6	ChCl/urea	1.0	35	38
7	ChCl/Gly	1.0	35	11
8	ChCl/urea	1.0^d^	35	35
9	ChCl/urea	1.0	50	62
10	ChCl/urea	1.0	70	74
11	ChCl/urea	1.0	80	70
12	ChCl/urea	1.0	70	70^e^
13	ChCl/urea	1.2	70	74
14	ChCl/urea	0.5	70	40
15	ChCl/urea	0.25	70	10
16	ChCl/urea^f^	1.0	70	<5
17	ChCl/urea	1.0	70	70^g^^,^ h

a Reaction conditions: 500 mg DES per 0.5 mmol of **1a**; choline chloride (ChCl)/urea (1:2 mol·mol^–1^); ChCl/glycerol (Gly) (1:2 mol·mol^–1^); choline acetate (ChOAc)/urea (1:2 mol·mol^–1^); ChCl/ethylene glycol (EG) (1:2 mol·mol^–1^).

b Calculated by ^1^H NMR analysis of the crude reaction mixture using an internal standard technique (NMR internal standard: CH_2_Br_2_).

c NR = no reaction.

d KBH_4_ was used in place of NaBH_4_.

e Reaction time: 48 h.

f NaBH_3_CN was used in place of NaBH_4_.

g Isolated yield (see main text).

h Scale up conditions: 1.0 g of **1a** (6.8 mmol) and 257 mg (6.8 mmol) of NaBH_4_ per 5.0 g ChCl/urea.

**FIGURE 2:**
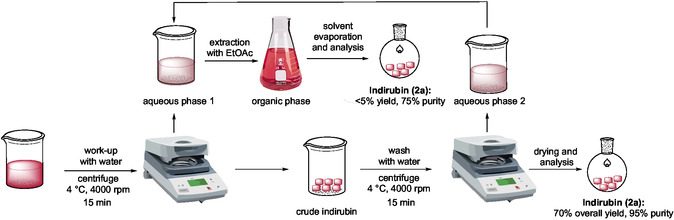
Work‐up procedure for the isolation of indirubin (**2a**).

With the 1.0 g‐scale indirubin synthesis optimized (Table 1, entry 17), we developed an efficient and sustainable chromatography‐free purification protocol that markedly minimized organic solvent use (Figure 2). This methodology advances sustainability by reducing reliance on VOCs and hazardous reagents, favoring a water‐based work‐up. After completion of the reaction, the crude mixture was allowed to cool to room temperature. Water was then added to the reaction, and the resulting biphasic mixture was transferred into a conical centrifuge tube and further cooled to 4°C to promote precipitation of the target compound. The first centrifugation was performed at 4000 rpm for 15 min at 4°C, effectively separating the mixture into a solid and a supernatant containing DES and aqueous phase. Following careful decantation of the supernatant, the solid residue was resuspended in a second aliquot of cold water and subjected to a second centrifugation under identical conditions to ensure efficient removal of residual DES and further improve product purity. The resulting supernatant was again decanted, and the combined aqueous fractions were extracted with EtOAc to recover any dissolved indirubin. Both the solid residue and the organic extracts were analyzed by ^1^H and ^13^C NMR, GC–MS, and HRMS to assess the purity and identity of the product. The purification protocol afforded indirubin (**2a**) in 70% overall isolated yield and 95% purity, as determined by ^1^H NMR analysis using CH_2_Br_2_ as an internal standard. In contrast, the indirubin recovered from the organic extracts accounted for less than 5% yield with a purity of 75%, and was therefore not further considered. For DES recovery, the aqueous phase obtained after EtOAc extraction was evaporated under reduced pressure with gentle heating until a constant weight of the DES was reached. The recovered DES appeared slightly darker and more viscous than the freshly prepared one. Upon reuse in a subsequent indirubin synthesis starting from 1.0 g (6.8 mmol) of **1a** and 257 mg (6.8 mmol) of NaBH_4_, compound **2a** was obtained in 40% yield (^1^H NMR analysis, CH_2_Br_2_ as an internal standard). Although a moderate decrease in yield was observed, this result clearly demonstrates the recoverability and reusability of the DES, further supporting the sustainability and circular potential of the process.

This streamlined work‐up procedure was specifically designed to minimize the environmental impact. The practicality and robustness of the optimized synthesis and purification protocol were further demonstrated by scaling up the reaction to a 5.0 g batch of **1a** (34 mmol) using NaBH_4_ (1.29 g, 34 mmol) in 25 g DES. Under the established conditions, indirubin **2a** was again successfully isolated in 70% yield (3.12 g, 11.9 mmol), without any appreciable loss in efficiency (Figure 2).

**FIGURE 3:**
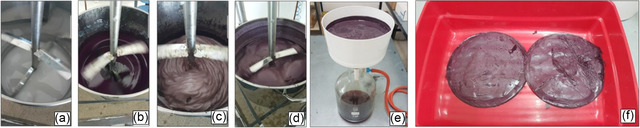
(a) Preparation of the ChCl/urea DES. (b) Reaction mixture after 2 h. (c) Reaction mixture after 24 h. (d) Appearance of the mixture following dilution with water. (e) Vacuum filtration of the crude reaction mixture. (f) Air‐drying of the collected solid on cellulose filters.

In partnership with Dieffetti Cosmetici S.r.l., based in Minervino Murge (Barletta‐Andria‐Trani, Italy) [58], we explored the pilot‐scale synthesis of compound **2a**. The reaction was carried out in ChCl/urea at 70°C for 24 h, employing a 1:1 molar ratio of isatin to NaBH_4_. Specifically, 386 g (2.62 mol) of isatin (**1a**) was added to 1.94 kg of pre‐prepared ChCl/urea eutectic mixture in the reaction reactor (Figure 3a) and stirred for 10 min to ensure complete dissolution. Then, 99.1 g (2.62 mol) of NaBH_4_ was added portionwise over the course of 1 h, under continuous stirring. The reaction mixture was maintained at 70°C under stirring for 24 h (Figure 3b,c). Upon completion, it was diluted with water (Figure 3d) and subsequently filtered under vacuum (Figure 3e; see the Supporting Information for further details). The resulting solid was collected, removed from the filtration funnel, and air‐dried (Figure 3f). The process afforded a 40% yield, which, although lower than that obtained on laboratory scale, successfully delivered 137.43 g of indirubin (**2a**) with a 95% purity, as determined by ^1^H NMR analysis using CH_2_Br_2_ as an internal standard. A truly promising outcome for a first pilot scale attempt at producing a pharmaceutical compound in a DES system.

**SCHEME 2:**
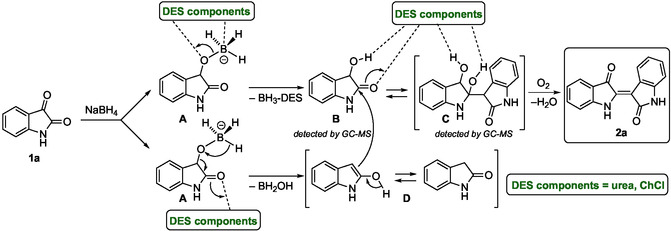
Plausible mechanism for the formation of indirubin (**2a**) by reductive dimerization of isatin (**1a**) with NaBH_4_ in a ChCl/urea mixture. Dashed lines indicate hydrogen‐bonding interactions with DES components.

A plausible mechanism for the formation of indirubin (**2a**) via reductive dimerization of isatin (**1a**) in the presence of NaBH_4_ is proposed and depicted in Scheme 2. The process begins with the reduction of **1a** by NaBH_4_, generating intermediate **A**. Within the DES environment, this intermediate may undergo elimination, leading to the formation of 3‐hydroxyisatin (**B**). Alternatively, **A** can undergo rearrangement, yielding 2‐hydroxyindole (**D**), in equilibrium with its tautomeric form. The resulting enolic species **D** then reacts with **B** to form adduct **C**. Finally, **C** undergoes an oxidative dehydration, in which aerial oxidation is accompanied by the elimination of water, affording the target compound **2a**. All these transformations are facilitated by the hydrogen‐bonding network of the DES, which likely plays a key role in stabilizing reactive intermediates, enhancing the electrophilicity and nucleophilicity of the species involved, and promoting proton transfer processes. The proposed mechanism is supported by the detection of intermediates **B** and **C** during the reaction, as confirmed by GC–MS and HRMS analyses (see the Supporting Information for details). Notably, the formation and reactivity of enolates in DES media have been previously reported [48], further supporting the plausibility of the proposed pathway.

To comprehensively evaluate the environmental performance of the synthetic route developed for the preparation of indirubin (**2a**), we applied a broad set of green chemistry metrics. These included Sheldon's E‐factor [56], along with key sustainability indicators such as atom economy (AE), reaction mass efficiency (RME), optimum efficiency (OE), effective mass yield (EM), and process mass intensity (PMI). Additionally, we calculated the renewables intensity (RI) and renewables percentage (RP) following the CHEM21 Metrics Toolkit developed by Clark and coworkers [57]. All metrics were determined on a 1.0 g scale (see the Supporting Information for details), and, where possible, compared with values reported for Liu's VOC‐based synthetic protocol (Table 2) [25]. Despite a lower yield (70% vs. 91%), the DES‐based method clearly outperforms the methanol‐based approach across several key green chemistry metrics. For instance, PMI_RXN_, which accounts for the chemicals and reaction solvents, was 10 (g·g^−1^) in our system, markedly lower than the 34.7 (g·g^−1^) reported for the methanol‐based synthesis. An even more striking improvement was observed in the EM, a metric that penalizes the use of toxic or hazardous inputs. The DES‐based route achieved an EM of 243%, representing a >80‐fold increase compared to its methanol‐based counterpart (3%), and reflecting a substantial reduction in the environmental burden associated with hazardous substances. Similarly, the E‐factor decreased from 33.7 in Liu's protocol [25] to 9 in our approach, highlighting a significantly lower amount of waste generated. Notably, the elevated PMI_WU_ (90.1) arises almost entirely from the nonrecycled aqueous work‐up used to isolate the product. While this increases the overall mass intensity, it does not reflect an intrinsic inefficiency of the transformation, which is better captured by a low PMI_RXN_ (10.0). Indeed, the work‐up mass consists predominantly of renewable and benign components (water and DES), as evidenced by the calculated RI (88.1) and RP (97.8) values, confirming the high contribution of renewable and low‐hazard materials.

**TABLE 2:** Quantitative metrics calculated for the procedures reported by Liu [25] and the present DES‐based approach for the synthesis of **2a**.

Reaction	Yield (%)	AE (%)	RME (%)	OE (%)	EM (%)	**PMI** **_RXN_** a (**g g** ^**–1**^ **)**	**PMI**_**WU**_ b **(g g** ^**–1**^ **)**	**RI** c	RP (%)	**E‐factor** d
Liu's procedure	91	75.3	68.3	90.7	3.0	34.7	ND^e^	ND^e^	ND^e^	33.7
Our approach	70	79.0	49.6	62.8	243.0	10.0	90.1^f^	88.1	97.8	9.0

a Process mass intensity (PMI)_RXN_: chemicals and reaction solvents.

b Process mass intensity (PMI)_WU_: chemicals and reaction solvents, solvents and reagents in work‐up.

c Renewable resources: DES, water.

d This value does not take into account the amount of solvent used during work‐up and/or purification steps such as centrifugation or crystallization.

e This value could not be determined.

f This value reflects the nonrecycled aqueous work‐up.

It is also worth highlighting that the sustainable synthetic route developed for the preparation of indirubin (**2a**) is not only environmentally benign but also economically advantageous. Based solely on the cost of raw materials, the estimated production cost amounts to €1.031 for 624 mg of indirubin (see the Supporting Information for detailed calculations). By comparison, the commercially available compound (CAS No. 906 748‐38‐7) is currently sold at a price of around €120 for just 5 mg, underscoring the remarkable cost‐efficiency of the proposed method [59].

**SCHEME 3:**
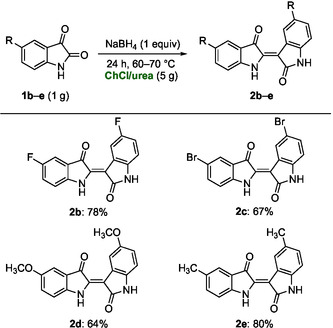
Synthesis of indirubin derivatives **2b**–**e** by reductive dimerization of isatin derivatives **1b**–**e** with NaBH_4_ in ChCl/urea.

The reproducibility and robustness of the methodology were further demonstrated through the sustainable synthesis of a series of disubstituted indirubins **2b**–**e**, bearing halogen, methyl, or methoxy groups at the 5 and 5′ positions of the aromatic rings. The derivatives obtained include: (2′*Z*)‐5,5′difluoroindirubin (**2b**), (2′*Z*)‐5,5′‐dibromoindirubin (**2c**), (2′*Z*)‐5,5′‐dimethoxyindirubin (**2d**), and (2′*Z*)‐5,5′‐dimethylindirubin (**2e**). All compounds were synthesized from the corresponding substituted isatins **1b**–**e** on a 1.0 g scale under the optimized conditions reported in Table 1, entry 17 (Scheme 3). Notably, the electronic nature of the substituents did not significantly influence the reaction yields. All derivatives **2b**–**e** were isolated and purified following the same procedure used for the parent compound **2a**, further confirming the operational simplicity of the DES‐based protocol.

Attempts to extend the methodology to the synthesis of 6,6′‐disubstituted indirubin derivatives were unsuccessful. When the corresponding 6‐substituted isatins (6‐MeO, 6‐Br, and 6‐F) were subjected to the NaBH_4_ reduction step under the optimized conditions, complex mixtures of degradation products were obtained instead of the expected indirubin derivatives. Some of these products were identified as species derived from isatin decomposition, suggesting that these substrates are less stable under the applied reductive conditions. These findings indicate that the one‐pot synthetic protocol described above is well suited for the preparation of 5,5′‐disubstituted indirubins, whereas it is not generally applicable to other substitution patterns.

**SCHEME 4:**
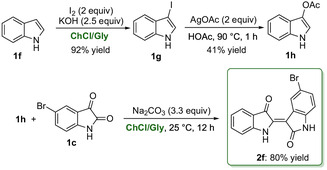
Synthesis of (2′*Z*)‐5‐bromoindirubin (**2f**) via 5‐bromoisatin (**1c**) in ChCl/Gly.

We further investigated the feasibility of synthesizing a monosubstituted indirubin, specifically (2′*Z*)‐5‐bromoindirubin (**2f**, Scheme 4), using DESs. This compound is of particular pharmacological interest due to its potent inhibitory activity against CDKs and glycogen synthase kinase‐3 (GSK‐3), both of which are key therapeutic targets in cancer, viral infections, and neurodegenerative diseases [60]. The precursor, 3‐acetoxyindole (**1h**), was prepared in two steps following the patented procedure by Dunn et al. [61], with a “green” modification involving the use of a ChCl/Gly eutectic mixture for the synthesis of 3‐iodoindole (**1g**) from indole (**1f**) [62]. In the final step, **1h** was coupled with 5‐bromoisatin (**1c**) in the same eutectic mixture, using Na_2_CO_3_ as a base. After 16 h stirring at room temperature, the crude product was purified by crystallization from an acetone/hexane mixture (hot‐to‐cold) (see the Supporitng Information for details) to afford **2f** in an 80% yield (Scheme 4).

**SCHEME 5:**
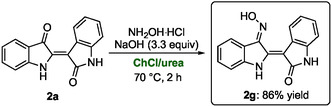
Synthesis of (2′*Z*)‐indirubin‐3′‐oxime (**2g**) in ChCl/urea.

Another indirubin derivative with potent inhibitory activity against CDKs, GSK‐3, and other kinases—making it a highly promising pharmacologically active compound—is (2′*Z*)‐indirubin‐3′‐oxime (**2g**, Scheme 5) [2]. Notably, **2g** has also been proposed for the treatment of Alzheimer's disease, and its hydroxamate‐substituted analogs have shown potential in anticancer therapy [5, 63, 64, 65]. This compound is commercially available from by Sigma‐Aldrich at € 825 per 5 mg (purity: >98%, HPLC) [66]. Conventional synthetic approaches to **2g** typically rely on VOCs such as pyridine and generally require reflux conditions above 100°C [2, 5].

In contrast, we have developed a DES‐based protocol using the environmentally benign eutectic mixture ChCl/urea at 70°C, in which **2a** reacts with hydroxylamine hydrochloride in the presence of NaOH, affording compound **2g** in 86% yield after just 2 h (Scheme 5). Importantly, the isolation and purification of **2g** were achieved without chromatography, by leveraging the acid–base properties of the oxime functionality. Upon reaction completion (monitored by TLC), the mixture was treated with 0.1 M aqueous HCl and extracted with EtOAc. The organic phase was then basified with 1 M NaOH, converting **2g** into its oximate salt, which was re‐extracted with EtOAc. The remaining aqueous phase was finally neutralized with 0.1 M aqueous HCl to pH 7 (monitored by litmus paper), prompting the precipitation of **2g** as a reddish solid in 86% yield with 98% chemical purity, as determined by ^1^H NMR analysis using CH_2_Br_2_ as an internal standard.

Molecules exhibiting photoswitching properties have long garnered significant attention from the scientific community due to their remarkable reactivity and functional versatility at the molecular level. Extensive research on classical photoswitch‐active scaffolds—such as stilbenes, azobenzenes, spiropyrans, and diarylethenes—has paved the way for a wide range of applications in molecular devices, smart materials, and biomedical systems [67, 68, 69, 70, 71]. Among indigoid compounds, indirubin can be converted into a red‐light‐responsive photoswitch via *N*‐alkylation of its NH protons. This modification not only enhances solubility, but also enables negative photochromism, in which the photoinduced isomerization can be reversed thermally or upon blue‐light irradiation [9].

Literature procedures for the synthesis of *N*‐monoalkylated and *N*,*N*′‐dialkylated indirubin derivatives typically rely on the use of DMF, a solvent increasingly restricted due to its toxicity, and NaH as a strong base for deprotonating the NH groups, followed by alkylation with alkyl halides, under an inert atmosphere [9]. In this work, we present a more sustainable and safer alternative using ChCl/urea as a DES and *t*‐BuOK as an easier‐to‐handle and less hazardous base in place of NaH. The optimized reaction parameters—including choice of DES, base, temperature, and reaction time—are summarized in Table 3. An excess of both base and electrophile was required to drive the reactions efficiently, even for monoalkylation. For instance, the use of 3 equiv of both *t*‐BuOK and propyl bromide led to a 93% yield of *N*‐propylindirubin (**3a**) (Table 3, entries 1,2). Higher reagent loadings were necessary to achieve dialkylation. However, when 4 equiv of each reagent were used, the reaction produced a 58:41 inseparable mixture of **3a** and *N*,*N*′‐dipropylindirubin (**4a**), as determined by ^1^H NMR and GC–MS analysis (Table 2, entry 3). This mixture was not further pursued. In contrast, the use of 4 equiv of both *t*‐BuOK and benzyl bromide enabled the selective formation and isolation of *N*,*N*′‐dibenzylindirubin (**4b**) in 60% yield (Table 2, entry 4) (see the Supporting Information for full experimental details).

**TABLE 3:** Synthesis of *N*‐propyl (**3a**) and *N*,*N*′‐dibenzyl (**4b**) indirubin derivatives in DESs.

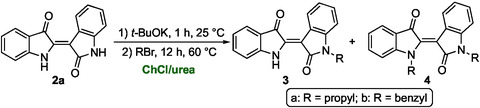				
Entry	*t*‐BuOK (equiv)	RBr (equiv)	Product **3** yield (%)^a^	Product **4** yield (%)^a^
1	2	CH_3_(CH_2_)_2_ (2)	**3a** (38)	**4a** (ND)^b^
2	3	CH_3_(CH_2_)_2_ (3)	**3a** (93)^c^	**4a** (<5%)
3	4	CH_3_(CH_2_)_2_ (4)	**3a** (58)^d^	**4a** (41)^d^
4	4	PhCH_2_Br (4)	**3b** (<5%)	**4b** (60)^c^

a Calculated by ^1^H NMR analysis of the crude reaction mixture using an internal standard technique (NMR internal standard: CH_2_Br_2_).

b ND = Not detected.

c Isolated yield.

d Inseparable mixture of **3a** and **4a** (^1^H NMR and GC–MS analysis).

## 3 Conclusion

We have developed an innovative, efficient, and sustainable method for the synthesis of indirubin (**2a**) by employing DESs as green reaction media. Systematic optimization identified the eutectic mixture of choline chloride/urea (1:2) as essential to promote both the reduction of isatin and the subsequent condensation step. Optimal results were achieved using a 1:1 molar ratio of isatin to NaBH_4_ at a reaction temperature of 70°C, which facilitated both the coupling of reactive intermediates and the final aerobic dehydration (Scheme 2), leading to **2a** in 70% overall yield up to a 5 g scale. A work‐up protocol minimizing the use of organic solvents was also developed, significantly reducing the environmental impact and improving operational safety. The methodology proved to be robust, generalizable, and operationally simple, enabling the efficient synthesis of various functionalized indirubin derivatives (**2b**–**e**) under identical conditions. Furthermore, pilot‐scale experiments involving 386 g of starting material in 1.94 kg of DES demonstrated the practical viability of this method for industrial implementation.

Furthermore, the use of eutectic mixtures was successfully extended to the synthesis of other biologically relevant indirubin derivatives, including 5‐bromoindirubin (**2f**), indirubin‐3′‐oxime (**2g**), as well as *N*‐propyl (**3a**) and *N*,*N*′‐dibenzyl (**4b**) analogs. These latter compounds are of interest due to their promising photoswitchable properties, thereby broadening the scope and potential applications of the developed strategy. The environmental performance of the process for the synthesis of **2a**, assessed through CHEM21 green chemistry metrics, revealed a 3.7‐fold reduction in the E‐factor and significantly improved EM and PMI values compared to conventional methanol‐based protocols. Notably, the DES‐based workflow eliminates chromatography for most indirubin derivatives and replaces hazardous solvents (e.g., methanol) with biodegradable, nonvolatile alternatives, thereby enabling a greener, safer, and scalable route to these pharmacologically valuable scaffolds.

## Supporting Information

Additional supporting information (experimental procedures, characterization details of the synthesized compounds including NMR spectra, green metrics calculation, Supporting Tables) can be found online in the Supporting Information section. The authors have cited additional references within the Supporting Information. [72–74] **Supporting** **Table** **S1:** Investigation of the synthesis of (2′*Z*)‐indirubin‐3′‐oxime (**2g**) from indirubin (**2a**) in different DES‐base systems. **Supporting** **Table** **S2:** Estimated cost for producing 624 mg of indirubin (**2a**), including reagents and solvents only, based on Sigma‐Aldrich prices (excluding analytical, energy, and labor costs).

## Funding

This work was supported by Ministero dell'Università e della Ricerca (2022KMS84P), European Union‐NextGenerationEU, Piano Nazionale di Ripresa e Resilienza (CUP H53D23004580006), Ministero dell’Ambiente e della Sicurezza Energetica (RSH2A_000004).

## Conflicts of Interest

The authors declare no conflicts of interest.

## Supporting information

Supplementary Material

## Data Availability

The data that support the findings of this study are available in the supplementary material of this article.
