# Supplementary material for: A Scalable and Sustainable Synthesis of Indirubin Frameworks Enabled by Deep Eutectic Solvents
Source: ChemSusChem. 2025 Dec 8;19(1):e202502114. doi: 10.1002/cssc.202502114 (PMC12767273; doi:10.1002/cssc.202502114)

Electronic Supporting information

Sustainable Synthesis of Indirubin Derivatives in Deep Eutectic Solvents: A Green and Scalable Approach

Biagio Delvecchio,[1] Luciana Cicco,[1] Andrea Nicola Paparella,[1] Gaetano Di Salvo,[2]

Filippo Maria Perna,[1,*] Vito Capriati[1,*]

[1] *Dipartimento di Farmacia–Scienze del Farmaco, Università degli Studi di Bari Aldo Moro, Consorzio CINMPIS, via E. Orabona 4, 70125 Bari,70125, Italy*

[2] *Dieffetti Cosmetici S.r.l., via G. Marconi, 76013 Minervino Murge (BT), Italy*

Table of Contents

1. General Methods and Materials S2

2. Experimental procedures S3

2.1 Synthesis of indirubin (**2a**) and related (2'*Z*)-5,5'-indirubin derivatives (**2b**–**e**): Typical procedure S3

2.2 Indirubin (**2a**) synthesis: scale-up reaction S3

2.3 Synthesis of (2'*Z*)-5-bromoindirubin (**2f**) S4

2.3.1 Synthesis of 3-iodoindole (**1g**) S4

2.3.2 Synthesis of 3-acetoxyindole (**1h**) S4

2.3.3 Synthesis of (2'*Z*)-5-bromoindirubin (**2f**) S4

2.4 Synthesis of (2'*Z*)-indirubin-3'-oxime (**2g**) S5

2.5 Synthesis of (2'*Z*)-*N*-propylindirubin (**3a**) S5

2.6 Synthesis of (2'*Z*)-*N,N'*-dibenzylindirubin (**4b**) S6

2.7 Table S1. Investigation of the synthesis of (2'*Z*)-indirubin-3'-oxime (**2g**) from indirubin (**2a**) in different DES-base systems S6

3. E-factor calculation for the synthesis of indirubin (2a) S7

4. Green metrics calculations for the synthesis of indirubin (2a) S8

5. Estimated cost for the synthesis of indirubin (2a) S12

6. Spectroscopic data for compounds 2a–g, 3a, 4b, and intermediates B and C S13

7. ^{1}H and ^{13}C NMR spectra S17

1. General Methods and Materials

For ^{1}H NMR (600 MHz) and ^{13}C NMR (150 MHz) analyses, $CDCl_3$ and DMSO-d_6 were used as solvents; chemical shifts are reported in parts per million (δ). FT-IR spectra were recorded on a Perkin-Elmer 681 spectrometer. Analytical thin-layer chromatography (TLC) was performed on pre-coated 0.25 mm plates of Kieselgel 60 F_{254}; visualization was accomplished under UV light (254 nm) or by spraying a solution of 5 % (w/v) ammonium molybdate and 0.2 % (w/v) cerium (III) sulfate in 17.6% (w/v) aq. sulfuric acid, following by heating to 473 K until blue spots appeared. GC-MS analyses were performed on a HP 5995C model. EtOAc was used as the extraction solvent during work-up, while water was employed for product purification, either by washing (liquids) or crystallization (solids). An Eppendorf 5180R centrifuge was used for product isolation. High-resolution mass spectrometry (HRMS) analyses were carried out using a Bruker microTOF QII mass spectrometer equipped with an electrospray ion source (ESI). Full characterization data, including HRMS analyses and copies of the ^{1}H and ^{13}C NMR spectra, are provided only for the newly synthesized compounds. The following abbreviations indicate signal multiplicities: s = singlet, d = doublet, m = multiplet, td = triplet of doublets. Unless otherwise specified, all reagents and solvents were purchased from Sigma-Aldrich (SigmaAldrich, St. Louis, MO, USA) and used without further purification. Deep Eutectic Solvents (DESs) [choline chloride (ChCl)/urea (1:2 mol/mol^{-1}); ChCl/glycerol (Gly)/ (1:2 mol/mol^{-1}); ChCl/L-lactic acid (LA) (1:1 mol/mol^{-1}); choline acetate (ChOAc)/urea (1:2 mol/mol^{-1}); ChCl/ethylene glycol (EG) (1:2 mol/mol^{-1})] were prepared by heating the corresponding components at 60–80 °C under stirring for 30–60 min until a clear solution was obtained.

2. Experimental procedures

2.1 Synthesis of indirubin (2a) and related (2'*Z*)-5,5'-indirubin derivatives (2b–e): Typical procedure

In a 50 ml vial, ChCl/urea (1:2 mol/mol, 5.0 g) and isatin (**1a**) (1 g, 6.8 mmol) were stirred at 70 °C for 10 min. Sodium borohydride ($NaBH_4$, 6.8 mmol, 257 mg) was then added slowly in portions. The reaction mixture was stirred at 70 °C for 24 h. After completion, the reaction mixture was cooled to room temperature, and water (25 mL) was added. The resulting mixture was transferred to a conical centrifuge tube, cooled in an ice bath for 10 min to induce precipitation, and then centrifuged at 4000 rpm and 4 °C for 15 min. Two distinct phases were obtained: a clear aqueous supernatant and a red solid at the bottom. The supernatant was carefully decanted, and the solid was resuspended in water (25 mL) and centrifuged again under the same conditions. After decantation, the combined aqueous layers were extracted with EtOAc (50 mL). The organic phase was dried over Na_2SO_4, filtered, and concentrated under reduced pressure. Both the solid residue and the organic extracts were analyzed by ^{1}H and ^{13}C NMR, GC-MS, and HRMS to assess the purity and identity of the product. Purification afforded indirubin (**2a**) in 70% overall isolated yield (624 mg) and 95% purity, as determined ^{1}H NMR analysis using CH_2Br_2 as an internal standard. In contrast, the indirubin recovered from the organic extracts was obtained in < 5% yield and 75% purity, and was therefore not further considered. For DES recovery, the aqueous phase obtained after EtOAc extraction was evaporated under reduced pressure with gentle heating until a constant weight of the DES was reached. The recovered DES appeared slightly darker and more viscous than the freshly prepared one. Upon reuse in a subsequent indirubin synthesis from 1.0 g (6.8 mmol) of **1a** and 257 mg (6.8 mmol) of $NaBH_4$, compound **2a** was obtained in 40% yield (^{1}H NMR analysis, CH_2Br_2 as an internal standard).

A reaction performed on a 5.0 g (34 mmol) scale of **1a**, according to the above procedure, employing $NaBH_4$ (1.29 g, 34 mmol), ChCl/urea (25 g), and water (2 × 125 mL), afforded **2a** in 70% yield (3.12 g, 11.9 mmol).

2.2 Indirubin (2a) synthesis: scale-up reaction

Isatin (**1a**) (2.62 mol, 386 g) was added to a pre-weighed ChCl/urea mixture (1:2 mol/mol, 1.94 kg) and stirred for 10 min to allow complete dissolution. Sodium borohydride ($NaBH_4$, 2.62 mol, 99.1 g) was then added in small aliquots at 15-min intervals. The reaction mixture was subsequently heated to 70 °C and stirred for 24 h. After this period, the reaction mixture was diluted with water (40 L). The addition of water under stirring led to the formation of foam. To allow its dissolution, the mixture was maintained under gentle agitation at room temperature for additional 24 h. Afterward, the mixture was filtered under vacuum using a Whatman grade 5 cellulose filter placed in a 24 cm diameter

Büchner funnel connected to a 10 L vacuum flask. Vacuum was generated using a VALUE VSV-20 peristaltic vacuum pump (20 m^3/h, final vacuum 0.5 mbar). To protect the pump from vapors generated during filtration, a Drechsel bottle filled with ice was inserted in-line between the vacuum flask and the pump. At the end of the filtration, the filter was removed from the funnel and air-dried. The retained solid was then scraped off, weighed, and analyzed by NMR, affording indirubin (**2a**) in 40% yield (137.43 g) and 95% purity as determined ^{1}H NMR analysis using CH_2Br_2 as an internal standard.

2.3 Synthesis of (2'*Z*)-5-bromoindirubin (2f)

2.3.1 Synthesis of 3-iodoindole (1g)

In a 100 mL round-bottom flask wrapped in aluminum foil and equipped with a magnetic stirrer, indole (**1f**) (1 mmol, 117 mg) was introduced. Iodine (2 mmol, 508 mg) and KOH (2.5 mmol, 140.3 mg) were then added, followed by ChCl/ethylene glycol (EG) (1:2 mol/mol, 2.5 g) as the solvent. A septum was placed on the flask, and the reaction mixture was stirred at 40 °C. Reaction progress was monitored by TLC (hexane:EtOAc = 8:2), confirming complete consumption of the starting material after 2 h. Upon completion, EtOAc (6 mL) was added to the reaction mixture, and the resulting suspension was filtered on celite to remove excess iodine. The organic solvent was concentrated under reduced pressure. The crude product, 3-iodoindole (**1g**), was obtained in 92% yield (224 mg) and 95% purity as determined ^{1}H NMR analysis using CH_2Br_2 as an internal standard. To preserve product integrity, argon was bubbled through the solution, the flask was stored in the freezer, and the product was used for the next step without further purification.

2.3.2 Synthesis of 3-acetoxyindole (1h)

In a 100 mL round-bottom flask equipped with magnetic stirring, 3-iodoindole (**1g**) (0.85 mmol, 206 mg) was dissolved in acetic acid (3 mL). Silver acetate (1.7 mmol, 284 mg) was then added, and the reaction mixture was heated at reflux (90 °C) for 2 h. Upon completion, the mixture was filtered through filter paper, and the solvent was removed under reduced pressure. The residue was treated with a saturated solution of sodium carbonate and brine to facilitate phase separation, followed by extraction with EtOAc (3×10 mL) The organic layer was concentrated under vacuum to afford 3-acetoxyindole (**1h**) in 41% yield (61 mg).

2.3.3 Synthesis of (2'*Z*)-5-bromoindirubin (2f)

In a 50 mL two-neck round-bottom flask equipped with a magnetic stir bar, 5-bromoisatin (**1c**) (0.5 mmol, 113 mg) and 3-acetoxyindole (**1h**) (0.5 mmol, 88 mg) were dissolved in anhydrous

ChCl/glycerol (Gly) (1:2 mol/mol, 2 mL). Sodium carbonate (1.65 mmol, 175 mg) was then added. The flask was sealed with septa on both necks, and the reaction mixture was subjected to three evacuation/nitrogen refill cycles (10 min under vacuum followed by 2 min under nitrogen) to ensure an inert atmosphere. The mixture was stirred at 25 °C for 16 h in the dark. Upon completion, the crude reaction mixture was dissolved in acetone (ca. 20 mL), and any insoluble particulates were removed by filtration at room temperature. The corresponding solution was then heated to 60 °C and allowed to cool slowly to room temperature. Hexane was subsequently added dropwise until turbidity appeared, and the mixture was placed in a –20 °C freezer for 12 h to induce crystallization of (2'*Z*)-5-bromoindirubin (**2f**) (80% yield, 131 mg).

2.4 Synthesis of (2'*Z*)-indirubin-3'-oxime (2g)

Indirubin (**2a**) (1g, 3.81 mmol) was added to a 100 mL round-bottom flask containing ChCl/urea (1:2 mol/mol, 5 g) and stirred for 10 min. Hydroxylamine hydrochloride (528 mg, 7.6 mmol) and NaOH (501 mg, 12.52 mmol) were then added. The flask was sealed with a rubber septum and the reaction mixture was heated at 70 °C for 2 h. After completion, the mixture was cooled to room temperature, and 30 mL of 0.1 M HCl were added. The resulting mixture was extracted with EtOAc (3×20 mL), and the organic phase was dried over Na_2SO_4, filtered, and evaporated under reduced pressure. The residue was then basified with 1 M NaOH and extracted again with EtOAc (3×20 mL). The remaining aqueous phase was neutralized with 0.1 M HCl to pH 7, and subjected to a final extraction with EtOAc (3×20 mL). The combined organic layers were dried over anhydrous Na_2SO_4, filtered, and concentrated under reduced pressure. (2'*Z*)-Indirubin-3'-oxime (**2g**) was obtained in 86% yield (0.91 g) and 98% purity as determined ^{1}H NMR analysis using CH_2Br_2 as an internal standard.

2.5 Synthesis of (2'*Z*)-*N*-propylindirubin (3a)

Indirubin (**2a**) (100 mg, 0.38 mmol) was placed in a 3 mL vial containing ChCl/urea (1:2 mol/mol, 500 mg) and stirred for 10 min at room temperature. Potassium *tert*-butoxide (*t*-BuOK, 128 mg, 1.14 mmol) was then added, and the resulting mixture was stirred for 1 h. Subsequently, 1-iodopropane (111 µL, 1.14 mmol) was added, the vial was sealed with a rubber septum, and the reaction was heated at 60 °C for 12 h. After completion, the reaction mixture was diluted with water (10 mL) and extracted with EtOAc (3×10 mL). The combined organic layers were dried over Na_2SO_4, filtered, and concentrated under reduced pressure. The crude product was purified by crystallization: it was dissolved in acetone at 60 °C, and hexane was slowly added until turbidity appeared. The suspension was stored at –20 °C for 12 h, affording (2'*Z*)-*N*-propylindirubin (**3a**) as a solid in 93% yield (107 mg).

2.6 Synthesis of (2'*Z*)-*N,N'*-dibenzylindirubin (4b)

Indirubin (**2a**) (50 mg, 0.19 mmol) was added to a 3 mL vial containing ChCl/urea (1:2 mol/mol, 380 mg) and stirred for 10 min. Potassium *tert*-butoxide (*t*-BuOK, 85.3 mg, 0.76 mmol) was then added, and the reaction mixture was stirred at room temperature for 1 h. Subsequently, benzyl bromide (68 μL, 0.57 mmol) was added, the vial was sealed with a rubber septum, and the reaction was heated at 60 °C for 16 h. After completion, the mixture was diluted with water (10 mL) and extracted with EtOAc (3×5 mL). The organic layer was dried over Na_2SO_4, filtered, and concentrated under reduced pressure. The crude residue was purified by column chromatography on silica gel using a hexane/EtOAc (12:1) mixture as eluent, affording (2'*Z*)-*N,N'*-dibenzylindirubin (**4b**) in 60% yield (51 mg).

2.7 Table S1. Investigation of the synthesis of (2'*Z*)-indirubin-3'-oxime (2g) from indirubin (2a) in different DES-base systems

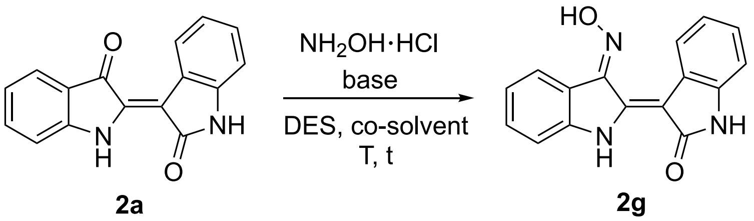

Entry	DES	Co-solvent (%)	Base (mmol)	$NH_3OH^+Cl^-$ (mmol)	t (h)	T (°C)	Conversion (%)[b]
1	ChCl/LA (1:1 mol mol^{-1})	–	$NaHCO_3$ (1.9)	8.12	24	110	50
2	ChCl/urea (1:2 mol mol^{-1})	–		0.43	16	110	NR[c]
3	"	EtOH (50)	NaOAc (2.66)	8.12	24	70	NR[c]
4	"	–	Et_3N (2.66)	8.12	24	75	NR[c]
5	"	–	NaOH (1.25)	0.76	2	70	86

[a] General conditions: reactions carried out with indirubin (**2a**, 0.19 mmol) in various deep eutectic solvents (DESs, 380 mg) using different bases; ChCl = choline chloride; LA = L-lactic acid. [b] Determined by ^{1}H NMR analysis of the crude reaction mixture using an internal standard technique (NMR internal standard: CH_2Br_2). [c] NR = no reaction.

3. E-factor calculation for the synthesis of indirubin (2a)

According to its original definition (*Green Chem*. **2007**, *9*, 1273), the Sheldon E–factor (total mass of waste/mass of isolated product) accounts solely for the mass of waste generated in a process. It is defined as the ratio between the mass of waste (calculated as the total mass of raw materials employed minus the mass of the desired isolated product) and the mass of the desired isolated product, based on the stoichiometric reaction equation. Accordingly, the amounts of silica gel, Celite, drying agents, and the solvents used for chromatography or crystallization are typically excluded from the calculation. We have adopted this definition and equation in our own determinations.

Classical synthesis of indirubin (2a)

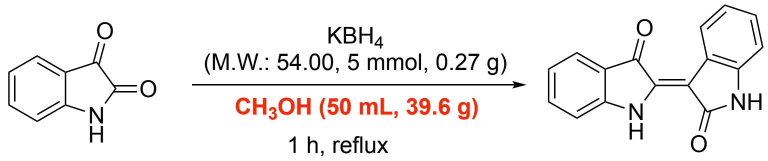

1a
1.471 g
M.W.: 147.133
10 mmol

2a: 91% yield
1.19 g
M.W.: 262.268
4.55 mmol

WORK -UP

Indirubin was isolated by filtration and further purified by recrystallization from 95% EtOH.

Total amount of reactants and reagents: 1.471 g (**isatin**) + 39.6 g (**CH_3OH**) + 0.27 g (**KBH_4**) = 41.341 g

Amount of the final product: 1.19 g

Amount of waste: 41.341 g – 1.19 g = 40.151 g

E-factor: amount of waste/amount of product = 40.151/1.19 g = **33.7**

Eco-friendly synthesis of indirubin (2a)

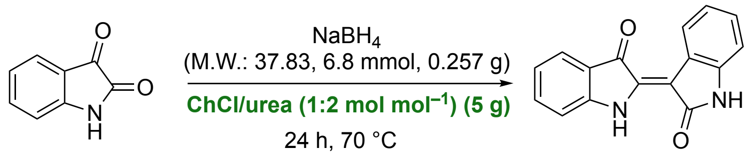

1a
1.0 g
M.W.: 147.133
6.8 mmol

2a: 70% yield
0.624 g
M.W.: 262.268
2.38 mmol

WORK -UP

The product was isolated by centrifugation and decantation of the aqueous phase as a purple solid.

Total amount of reactants: 1 g (**isatin**) + 5 g (**DES**) + 0.257 g (**$NaBH_4$**) = 6.257 g

Amount of the final product: 0.624 g

Amount of waste: 6.257 g – 0.624 g = 5.633 g

E-factor: amount of waste/amount of product = 5.633 g/0.624 g = **9.0**

4. Green metrics calculations for the synthesis of indirubin (2a)[1]

Metric	Acronym	Formula
Atom economy	AE	$\frac{\text{m. w. product}}{\Sigma \text{ m. w. reagents}} \times 100$
Reaction mass efficiency	RME	$\frac{\text{mass of product (kg)}}{\Sigma \text{ mass reagents (kg)}} \times 100$
Effective mass yield	EM	$\frac{\text{mass of products (kg)}}{\text{mass of non-benign reagents (kg)}} \times 100$
Optimum efficiency	OE	$\frac{\text{RME}}{\text{AE}} \times 100$
Process mass intensity	PMI	$\frac{\text{total mass in a process}}{\text{mass of product}}$
Renewables intensity	RI	$\frac{\text{mass of all renewably derivable materials used}}{\text{mass of product}}$
Renewables percentage	RP	$\frac{\text{RI}}{\text{PMI}} \times 100$

[1] (a) C. R. McElroy, A. Constantinou, L. C. Jones, L. Summerton, J. H. Clark, *Green Chem.* **2015**, *17*, 3111–3121; (b) S. Abou-Shehada, P. Mampuys, B. U. W. Maes, J. H. Clark, L. Summerton, *Green Chem.* **2017**, *19*, 249–258.

Quantitative comparison of conventional and eco-friendly approaches to the synthesis of indirubin (2a)

Conventional synthesis of indirubin (2a)

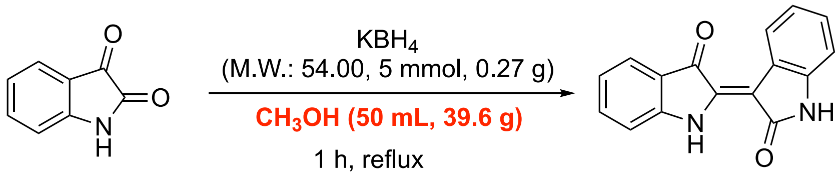

1a
1.471 g
M.W.: 147.133
10 mmol

2a: 91% yield
1.19 g
M.W.: 262.268
4.54 mmol

WORK -UP

The solid was isolated by filtration and was further purified by recrystallization from 95% EtOH.

Detailed Solvent: CH_3OH: highly flammable and hazardous

Eco-friendly synthesis of indirubin (2a)

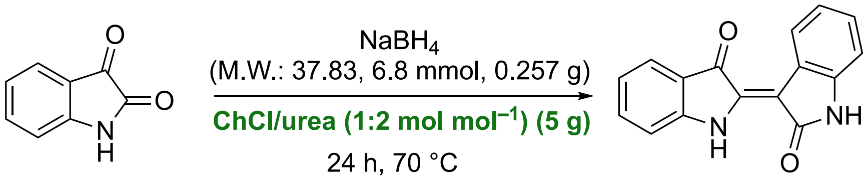

1a
1.0 g
M.W.: 147.133
6.8 mmol

2a: 70% yield
0.624 g
M.W.: 262.268
2.38 mmol

WORK -UP

The product was isolated as a purple solid by centrifugation and subsequent decantation of the aqueous phase.

Detailed Solvent: DES: recommended

AE

Conventional synthesis of indirubin (2a)

M.W. Reactants: 147.133 × 2 (**isatin**) + 54.00 (**KBH_4**) = 348.266

AE: 262.268/348.266 × 100 = **75.3%**

Eco-friendly synthesis of indirubin (2a)

M.W. Reactants: 147.133 × 2 (**isatin**) + 37.83 (**$NaBH_4$**) = 332.096

AE: 262.268/332.096 × 100 = **79.0%**

RME

Conventional synthesis of indirubin (2a)

1.19 g/1.741 g × 100 = **68.3%**

Eco-friendly synthesis of indirubin (2a)

0.624 g/1.257 g × 100 = **49.6%**

OE

Conventional synthesis of indirubin (2a)

68.3/75.3 × 100 = **90.7%**

Eco-friendly synthesis of indirubin (2a)

49.6/79.0 × 100 = **62.8%**

EM

Conventional synthesis of indirubin (2a)

Mass of non benign reagents: 39.6 g (**MeOH**) + 0.27 g (**KBH_4**) = 39.87 g

EM: 1.19 g/39.87 g × 100 = **3.0%**

Eco-friendly synthesis of indirubin (2a)

Mass of non benign reagents: 0.257g (**$NaBH_4$**)

EM: 0.624 g/0.257g × 100 = **243.0%**

PMI

Conventional synthesis of indirubin (2a)

Total amount of reactants: 1.471 g (**isatin**) + 39.6 g (**MeOH**) + 0.27 g (**KBH_4**) = 41.341 g

Amount of the final product: 1.19 g

PMI_{RXN}[a] 41.341 g/1.19 g = **34.7** (g g^{-1})

PMI_{WU}[b] ND

Eco-friendly synthesis of indirubin (2a)

Total amount of reactants: 1 g (**isatin**) + 5 g (**DES**) + 0.257 g (**$NaBH_4$**) = 6.257 g

Amount of the final product: 0.624 g

PMI_{RXN}[a] 6.257 g/0.624 g = **10.0** (g g^{-1})

PMI_{WU}[b] [50 g (water) + 6.257 g]/0.624 g = **90.1** (g g^{-1})

[a] Process mass intensity $(PMI)_{RXN}$: chemicals and reaction solvents.

[b] Process mass intensity $(PMI)_{WU}$: chemicals and reaction solvents, solvents, and reagents in workup.

RI and RP

Conventional synthesis of indirubin (2a)

Renewable sources:

RI: ND RP: ND

Eco-friendly synthesis of indirubin (2a)

Renewable sources: = 5 g (**DES**) + 50 g (**H_2O**) = 55

RI: 55 g/0.624 g = **88.1** **RP**: 88.1/90.1 × 100 = **97.8%**

5. Estimated cost for the synthesis of indirubin (2a)

Table S2. Estimated cost for producing 624 mg of indirubin (2a), including reagents and solvents only, based on Sigma-Aldrich prices (excluding analytical, energy, and labor costs)

Reactant/Reagent/Solvent	Quantity	Cost (€)
ChCl/urea	5 g	0.622
isatin	1 g	0.222
$NaBH_4$	0.257 g	0.107
H_2O	50 mL	0.08
Total production cost for 624 mg of indirubin		**1.031**

6. Spectroscopic data of compounds 2a–g, 3a, 4b, and intermediates B and C

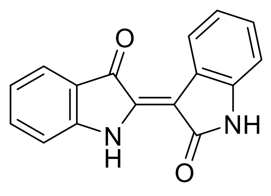

(2'*Z*)-Indirubin (**2a**)[2]: (624 mg from 1 g of **1a**, 70% yield), purple-red solid, purified by centrifugation and decantation, mp = 351–353 °C; ^{1}H NMR (600 MHz, DMSO-d_6): δ 11.00 (s, 2 H), 8.77–8.76 (m, 1 H), 7.66–7.59 (m, 1 H), 7.58–7.56 (m, 1 H), 7.43–7.41 (m, 1 H), 7.27–7.24 (m, 1 H), 7.03–7.01 (m, 2 H), 6.91–6.90 (m, 1 H); ^{13}C NMR (150 MHz, DMSO-d_6): δ 188.6, 170.8, 159.5, 152.4, 140.8, 138.2, 137.0, 129.2, 124.6, 124.3, 121.3, 121.2, 118.9, 113.3, 109.5, 106.5; FT-IR (KBr, cm^{-1}): 3345, 3193, 1663, 1621, 1462, 1381, 1303, 1210, 1178, 1144, 1003, 750; GC MS (70 ev) *m/z* (rel. int.): 262 (M$^+$, 19), 234 (54), 205 (41), 177 (4), 158 (6), 131 (15), 117 (2), 103 (25), 76 (25), 51 (8); HRMS (ESI): *m/z* Calcd for $C_{16}H_9N_2O_2$ [M-H]$^-$ 261.0670, found: 261.0664.

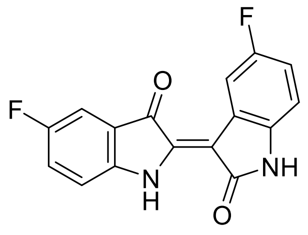

(2'*Z*)-5,5'-Difluoroindirubin (**2b**)[2]: (704 mg from 1 g of **1b**, 78% yield), purple-red solid, purified by centrifugation and decantation, mp = 289–291 °C; ^{1}H NMR (600 MHz, DMSO-d_6): δ 11.07 (s, 1 H), 10.90 (s, 1 H), 8.52–8.49 (m, 1 H), 7.47–7.43 (m, 3 H), 7.11–7.06 (m, 1 H), 6.89–6.84 (m, 1 H); ^{13}C NMR (150 MHz, DMSO-d_6): δ 188.8 (d, $^4J_{C\text{-}F}$ = 3.4 Hz), 171.2, 158.9 (d, $^1J_{C\text{-}F}$ = 238.8 Hz), 157.0 (d, $^1J_{C\text{-}F}$ = 233.3 Hz), 149.6, 139.9, 137.8, 124.7 (d, $^2J_{C\text{-}F}$ = 24.8 Hz), 122.8 (d, $^3J_{C\text{-}F}$ = 10.5 Hz), 119.9 (d, $^3J_{C\text{-}F}$ = 7.8 Hz), 115.8 (d, $^2J_{C\text{-}F}$ = 24.0 Hz), 115.5 (d, $^3J_{C\text{-}F}$ = 7.7 Hz), 111.7 (d, $^2J_{C\text{-}F}$ = 27.5 Hz), 110.8 (d, $^2J_{C\text{-}F}$ = 23.8 Hz), 110.6 (d, $^3J_{C\text{-}F}$ = 8.7 Hz), 106.9 (d, $^4J_{C\text{-}F}$ = 3.2 Hz); ^{19}F NMR (282 MHz, DMSO-d_6): δ -122.07 (td, $J_{F\text{-}H}$ = 9.9, 4.8 Hz), -122.36 (td, $J_{F\text{-}H}$ = 7.9, 5.0 Hz); FT-IR (KBr, cm^{-1}): 3336, 1669, 1629, 1595, 1484, 1279, 1190, 1146, 1018, 939, 821; GC MS (70 ev) *m/z* (rel. int.): 298 (M$^+$, 100), 270 (58), 241 (29), 213 (4), 195 (2), 176 (5), 149 (8), 121 (25), 94 (17), 75 (2); HRMS (ESI): *m/z* Calcd for $C_{16}H_7F_2N_2O_2$ [M-H]$^-$ 297.0481, found: 297.0473.

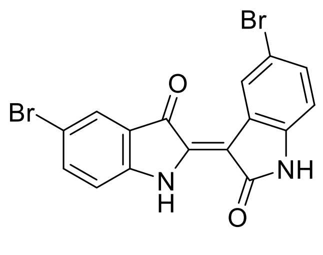

(2'*Z*)-5,5'-Dibromoindirubin (**2c**)[2]: (622 mg from 1 g of **1c**, 67% yield), purple-red solid, purified by centrifugation and decantation, mp = 299–301 °C; ^{1}H NMR (600 MHz, DMSO-d_6): δ 11.05 (s, 2 H), 8.91–8.87 (m, 1 H), 7.80–7.79 (m, 1 H), 7.75–7.71 (m, 1 H), 7.40–7.38 (m, 2 H), 6.86–6.84 (m, 1 H); ^{13}C NMR (150 MHz, DMSO-d_6): δ 187.6, 170.4, 151.4, 140.1, 139.3, 138.7, 131.5, 126.8, 126.6, 123.3, 120.8, 115.8, 113.2, 113.0, 111.4, 105.9; FT-IR (KBr,

[2] C. Wang, J. Yan, M. Du, J. A. Burlison, C. Li, Y. Sun, D. Zhao, J. Liu, *Tetrahedron* **2017**, *72*, 2780–2785.

cm^{-1}): 3487, 3100, 1675, 1613, 1464, 1289, 1211, 1123, 1015, 818; GC-MS (70 ev) *m/z* (rel. int.): 422 (M^+ + 4, 50), 420 (M^++2, 100), 418 (M^+, 61), 391 (6), 362 (5), 340 (8), 313 (15), 283 (38), 253 (5), 223 (5), 204 (10), 177 (14), 156 (5), 129 (3), 102 (13); HRMS (ESI): *m/z* Calcd for $C_{16}H_7Br_2N_2O_2$ $[M-H]^-$ 416.8880, found: 416.8876.

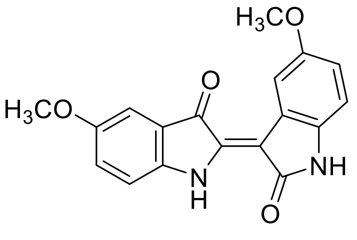

(2'*Z*)-5,5'-Dimethoxyindirubin (**2d**)[3]: (582 mg from 1 g of **1d**, 64% yield), blue solid, purified by centrifugation and decantation, mp = 258–260 °C; ^{1}H NMR (600 MHz, DMSO-*d$_6$*): δ 10.82 (s, 1 H), 10.65 (s, 1 H), 8.45–8.44 (m, 1 H), 7.33–7.30 (m, 1 H), 7.19–7.18 (m, 1 H), 7.15–7.14 (m, 1 H), 6.84–6.81 (m, 1 H), 6.80–6.75 (m, 1H), 3.76 (s, 3 H), 3.75 (s, 3 H); ^{13}C NMR (150 MHz, DMSO-*d$_6$*): δ 188.8, 171.0, 154.5, 154.3, 147.3, 139.1, 134.7, 125.3, 122.2, 119.2, 114.9, 114.5, 110.3, 109.2, 106.8$_2$, 106.8$_0$, 56.7, 55.3; FT-IR (KBr, cm^{-1}): 3324, 3169, 2829, 1662, 1623, 1597, 1493, 1320, 1285, 1197, 1157, 1013, 890; GC-MS (70 ev) *m/z* (rel. int.): 322 (M^+, 100), 307 (21), 293 (3), 279 (26), 264 (2), 251 (10), 236 (2), 208 (4), 192 (2), 161 (5), 147 (1), 125 (1), 90 (2), 77 (1); HRMS (ESI): *m/z* Calcd for $C_{18}H_{13}N_2O_4$ $[M-H]^-$ 321.0881, found: 321.0887.

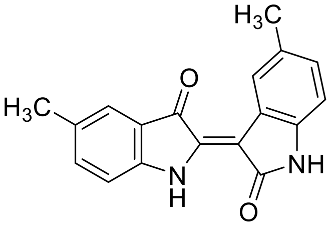

(2'*Z*)-5,5'-Dimethylindirubin (**2e**)[2]: (720 mg from 1 g of **1e**, 80% yield) purple-red solid, purified by centrifugation and decantation, mp = 308–310 °C; ^{1}H NMR (600 MHz, DMSO-*d$_6$*): δ 10.89 (s, 1 H), 10.78 (s, 1 H), 8.60 (s, 1 H), 7.43–7.40 (m, 1 H), 7.39–7.38 (m, 1 H), 7.30–7.29 (m, 1 H), 7.06–7.05 (m, 1 H), 6.79–6.78 (m, 1 H), 2.32 (s, 3 H), 2.29 (s, 3 H); ^{13}C NMR (150 MHz, DMSO-*d$_6$*): δ 188.7, 171.1, 150.7, 138.7, 138.6, 138.0, 130.4, 129.8, 129.7, 125.1, 124.1, 121.6, 119.2, 113.2, 109.3, 106.5, 21.1, 20.2; FT-IR (KBr, cm^{-1}): 3428, 3323, 3177, 2916, 2860, 1662, 1624, 1593, 1492, 1336, 1291, 1197, 1125, 1023, 984, 811; GC-MS (70 ev) *m/z* (rel. int.): 290 (M^+, 100), 262 (29), 247 (2), 219 (3), 191 (2), 145 (7), 123 (2), 116 (2), 103 (2); HRMS (ESI): *m/z* Calcd for $C_{18}H_{13}N_2O_2$ $[M-H]^-$ 289.0983, found: 289.0972.

---

[3] Y. Kosuge, H. Saito, T. Haraguchi, Y. Ichimaru, S. Ohashi, H. Miyagishi, S. Kobayashi, K. Ishige, S. Miyairi, Y. Ito, *Bioorg. Med. Chem. Lett.* **2017**, 27, 5122–5125.

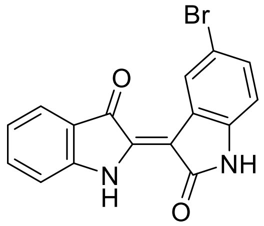

(2'*Z*)-5-Bromoindirubin (2f): (131 mg from 113 mg of **1g**, 80% yield) red solid, purified by crystallization from a hot-to-cold acetone/hexane mixture, mp >250 °C; ^{1}H NMR (600 MHz, DMSO-d_6) δ 11.13 (s, 1 H), 11.03 (s, 1 H), 8.95 (s, 1 H), 7.68–7.67 (m, 1 H), 7.62–7.59 (m, 1 H), 7.45–7.41 (m, 2 H), 7.07–7.05 (m, 1 H), 6.88–6.86 (m, 1 H); ^{13}C NMR (150 MHz, DMSO-d_6) δ 189.3, 171.0, 153.0, 140.3, 139.7, 137.8, 131.6, 127.1, 125.0, 124.0, 122.2, 119.5, 114.1, 113.4, 111.8, 105.4; FT-IR (KBr, cm^{-1}) 3425, 3335, 1697, 1662, 1614, 1590, 1480, 1465, 1384, 1212; HRMS (ESI): *m/z* Calcd for $C_{16}H_8BrN_2O_2$ [M−H]$^-$ 338.9775, found: 338.9781.

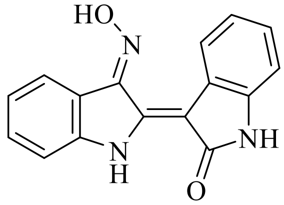

(2'*Z*)-Indirubin-3'-oxime (2g)[4]: (910 mg from 1 g of **2a**, 86% yield) purple red solid, isolated by acid-base washing followed by extraction with EtOAc, mp >250 °C; ^{1}H NMR (600 MHz, DMSO-d_6) δ 13.47 (s, 1 H), 11.74 (s, 1 H), 10.71 (s, 1 H), 8.66 (d, *J* = 7.8 Hz; 1 H), 8.25 (d, *J* = 7.7 Hz, 1 H), 7.41–7.38 (m, 2 H), 7.16–7.13 (m, 1 H), 7.05–7.03 (m, 1 H), 6.98–6.95 (m, 1 H), 6.92–6.91 (m, 1 H); ^{13}C NMR (150 MHz, DMSO-d_6) δ 171.5, 151.8, 145.8, 145.3, 138.8, 132.5, 128.5, 126.4, 123.5, 123.2, 121.9, 120.8, 117.0, 111.9, 109.3, 99.4; FT-IR (KBr, cm^{-1}) 3246, 3058, 1659, 1614, 1567, 1463, 1328, 1224, 1167, 747; HRMS (ESI): *m/z* Calcd for $C_{16}H_{12}N_3O_2$ [M+H]$^+$ 278.0924, found: 278.0933.

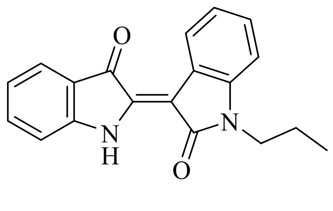

(2'*Z*)-*N*-Propylindirubin (3a)[5] : (107 mg from 100 mg of **2a**, 93% yield) purple solid, purified by crystallization from a hot-to-cold acetone/hexane mixture, mp = 171–173 °C; ^{1}H NMR (600 MHz, $CDCl_3$): δ 10.59 (s, 1 H), 8.92– 8.91 (m, 1 H), 7.76–7.74 (m, 1 H), 7.53–7.50 (m, 1 H), 7.33–7.27 (m, 1 H), 7.15–7.13 (m, 1 H), 7.03–7.01 (m, 1 H), 6.99–6.98 (m, 1 H), 6.91–6.90 (m, 1 H), 3.82 (t, *J* = 7.2 Hz, 2 H), 1.81–1.74 (m, 2 H), 1.01 (t, *J* = 7.3 Hz, 3 H); ^{13}C NMR (150 MHz, $CDCl_3$) δ 189.0, 171.5, 152.5, 142.3, 140.1, 137.6, 129.9, 126.4, 126.0, 123.2, 122.3, 122.1, 120.9, 112.6, 108.8, 107.5, 42.3, 21.9, 12.1; FT-IR (KBr, cm^{-1}) 3312, 3054, 2963, 2930, 2871, 1703, 1663, 1651, 1626, 1604, 1488, 1466, 1380, 1361, 1320, 1293, 1260, 1211, 1194, 1177, 1158, 1145, 1130, 1102, 1092; HRMS (ESI): *m/z* Calcd for $C_{19}H_{17}N_2O_2$ [M+H]$^+$ 305.1285, found: 305.1295.

---

[4] (a) X. Cheng, K. H. Merz, S. Vatter, J. Zeller, S. Muehlbeyer, *J. Med. Chem.* **2017**, *60*, 4949−4962; (b) R. Hoessel, S. Leclerc, J. A. Endicott, M. E. M. Nobel, A. Lawrie, P. Tunnah, M. Leost, E. Damiens, D. Marie, D. Marko, E. Niederberger, W. Tang, G. Eisenbrand, L. Meijer, *Nat. Cell Biol.* **1999**, 1, 60–67.

[5] S. Thumser, L. Köttner, N. Hoffmann, P. Mayer, H. Dube, *J. Am. Chem. Soc.* **2021**, *143*, 18251−18260.

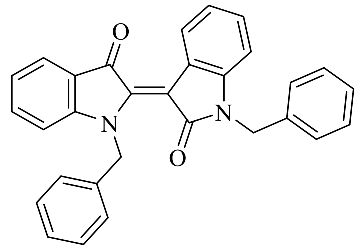

(2'*Z*)-*N,N'*-Dibenzylylindirubin (4b): (51 mg from 50 mg of **2a**, 60% yield) blue solid, purified by column chromatography (hexane/EtOAc 12:1), mp > 250 °C; ^{1}H NMR (600 MHz, DMSO-d_6): δ 8.65–8.64 (m, 1 H), 7.75–7.74 (m, 1 H), 7.48–7.45 (m, 1 H), 7.32–7.29 (m, 9 H), 7.25–7.15 (m, 1 H), 7.06–7.05 (m, 1 H), 7.02–6.97 (m, 1 H), 6.71–6.70 (m, 1 H), 5.60 (s, 2 H) 5.02 (s, 2 H); ^{13}C NMR (151 MHz, $CDCl_3$): δ 188.1, 167.0, 154.0, 142.4, 141.8, 136.4, 136.2, 136.1, 129.5, 128.7, 128.6, 127.5, 127.4, 127.3, 127.1, 125.5, 124.9, 122.4, 122.2, 121.8, 121.7, 112.4, 111.0, 108.5, 52.3, 43.7; FT-IR (KBr, cm^{-1}) 2924, 2853, 1738, 1668, 1604, 1550, 1467, 1329, 1170, 1083, 1016, 745; HRMS (ESI): *m/z* Calcd for $C_{30}H_{23}N_2O_2$ $[M+H]^+$ 443.1754, found: 443.1765.

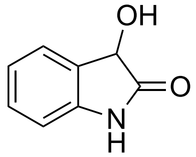

Intermediate B: GC-MS (70 ev) *m/z* (rel. int.): 149 (M^+, 62), 121 (18), 93 (100), 66 (22); HRMS (ESI): *m/z* Calcd for $C_8H_7NO_2$ $[M+Na]^+$ 172.0369, found 172.0360; $[M-H]^-$ 148.0404, found 148.0404.

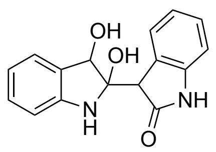

Intermediate C: GC-MS (70 ev) *m/z* (rel. int.): 282 (M^+, 12), 281 (18), 137 (91), 133 (100), 130 (85), 104 (90); HRMS (ESI): *m/z* Calcd for $C_{16}H_{14}N_2O_3$ $[M+Na]^+$ 305.0897, found: 305.0890; $[M-H]^-$ 281.0932, found: 281.0927.

7. ^{1}H and ^{13}C NMR spectra

^{1}H NMR, 600 MHz, DMSO-d_6 (**2a**)

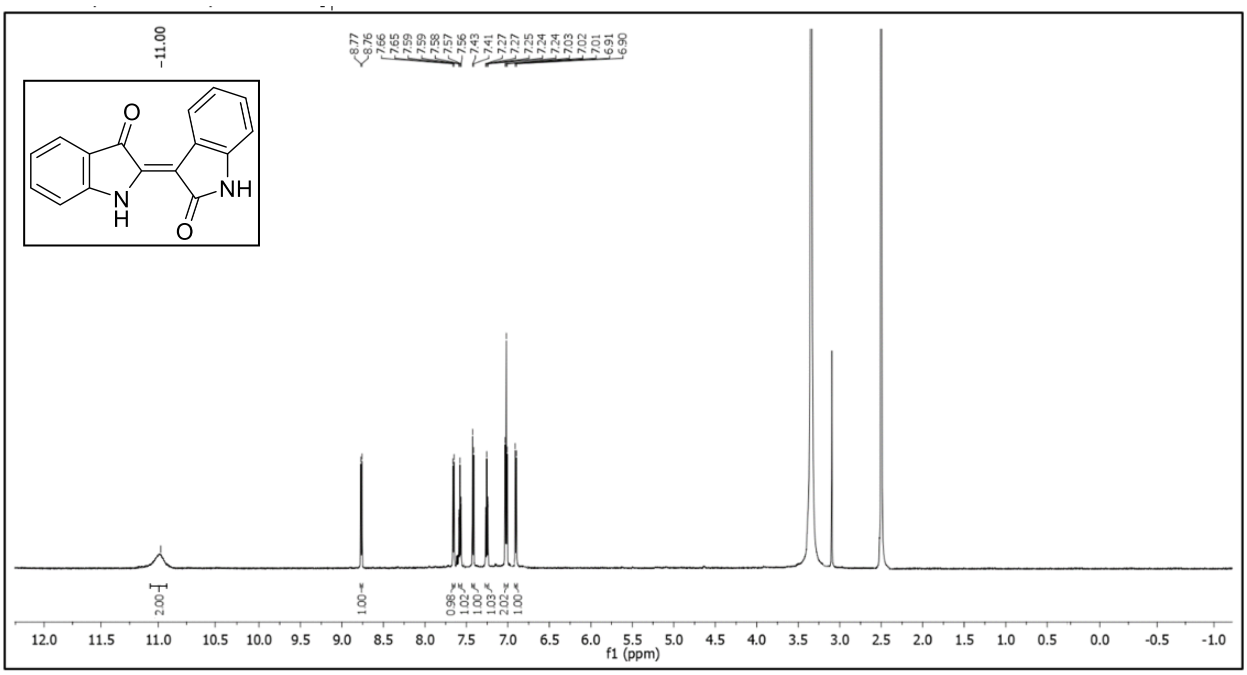

^{13}C NMR, 150 MHz, DMSO-d_6 (**2a**)

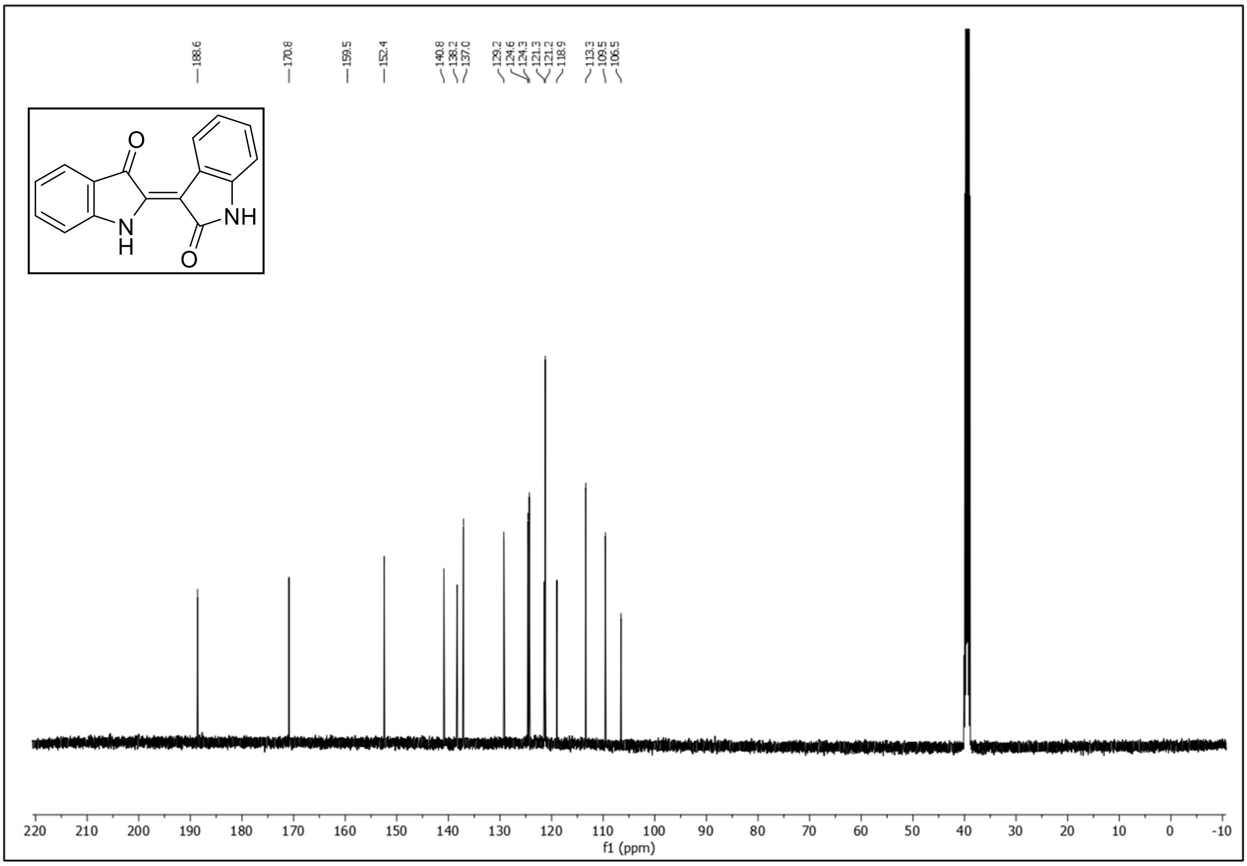

^{1}H NMR, 600 MHz, DMSO-d_6 (**2b**)

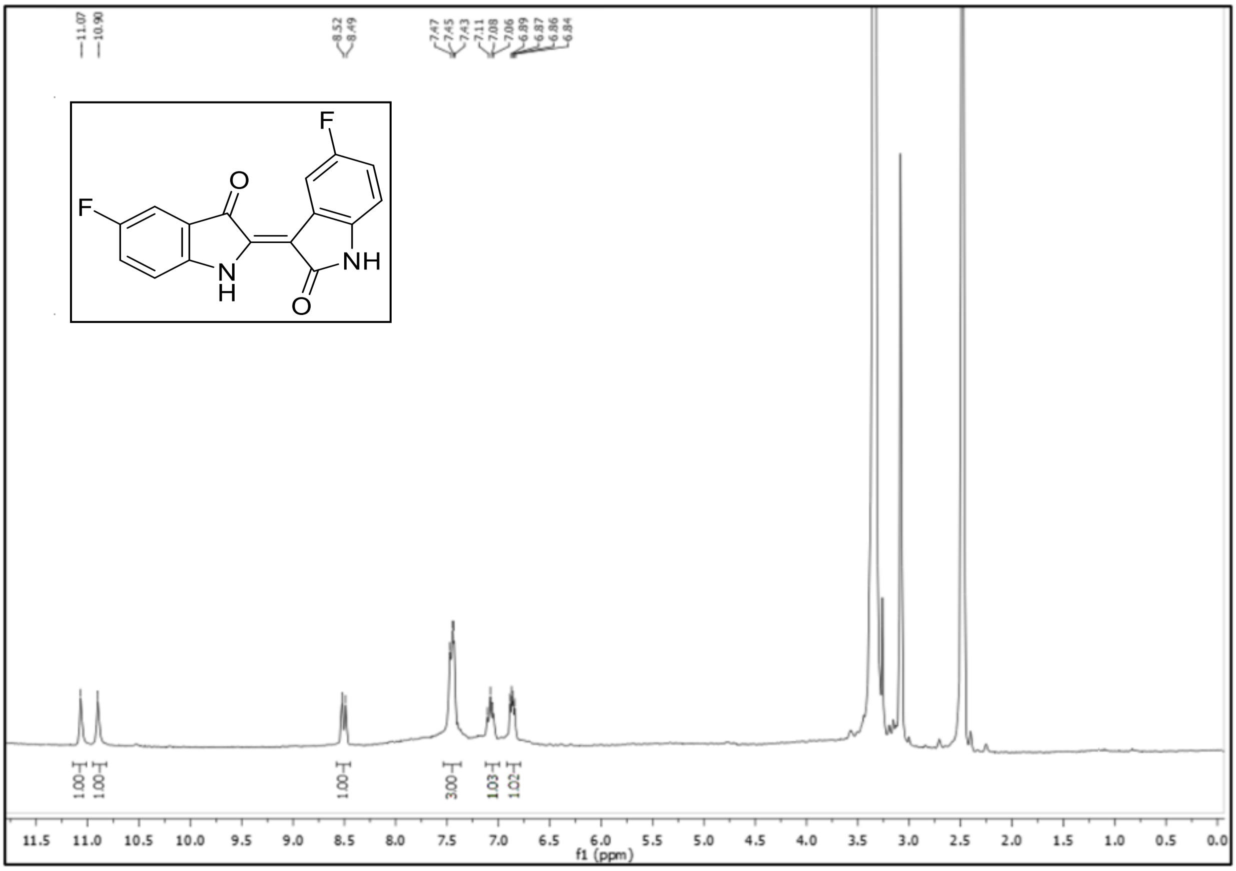

^{13}C NMR, 150 MHz, DMSO-d_6 (**2b**)

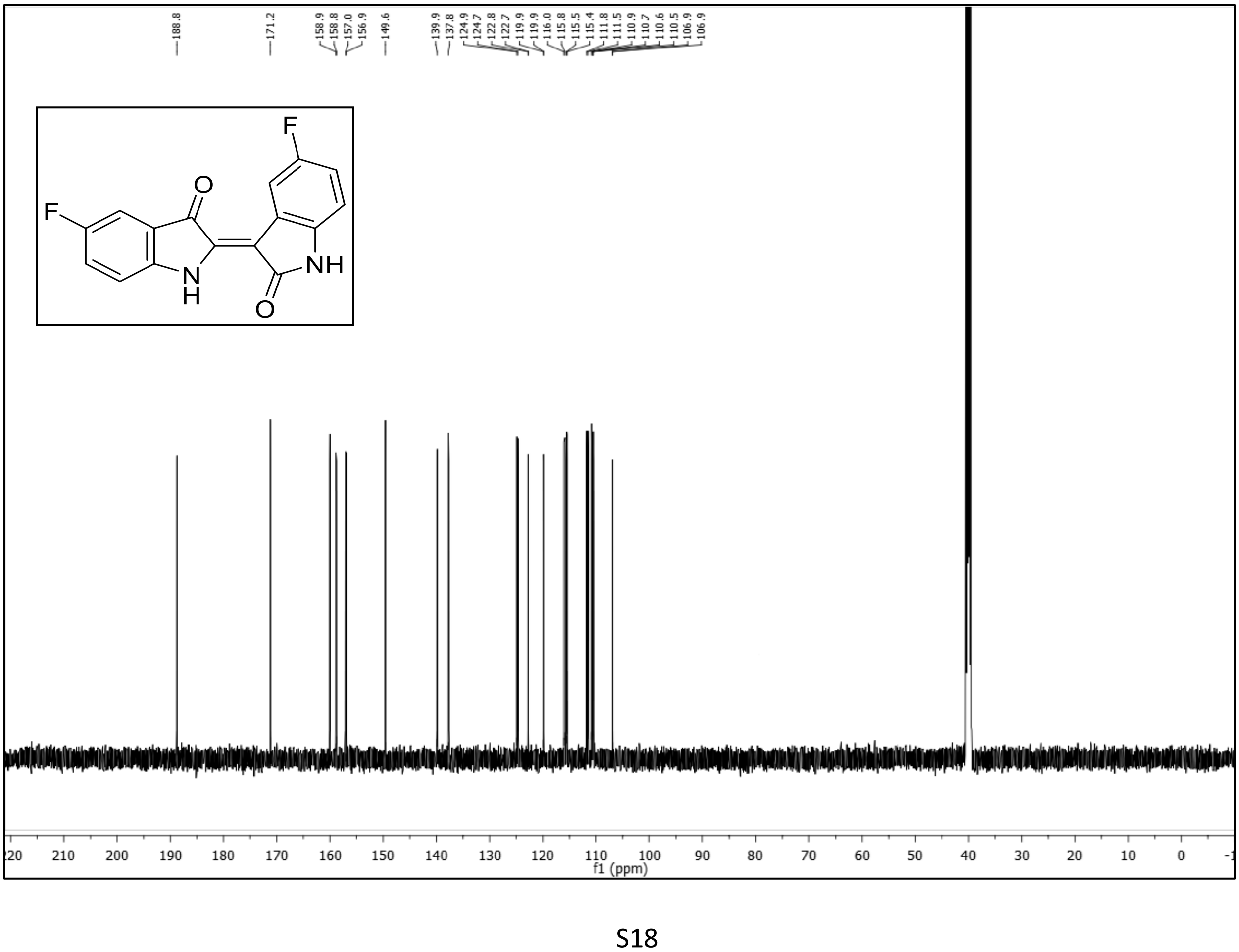

[19] F NMR, 150 MHz, DMSO-d_6 (**2b**)

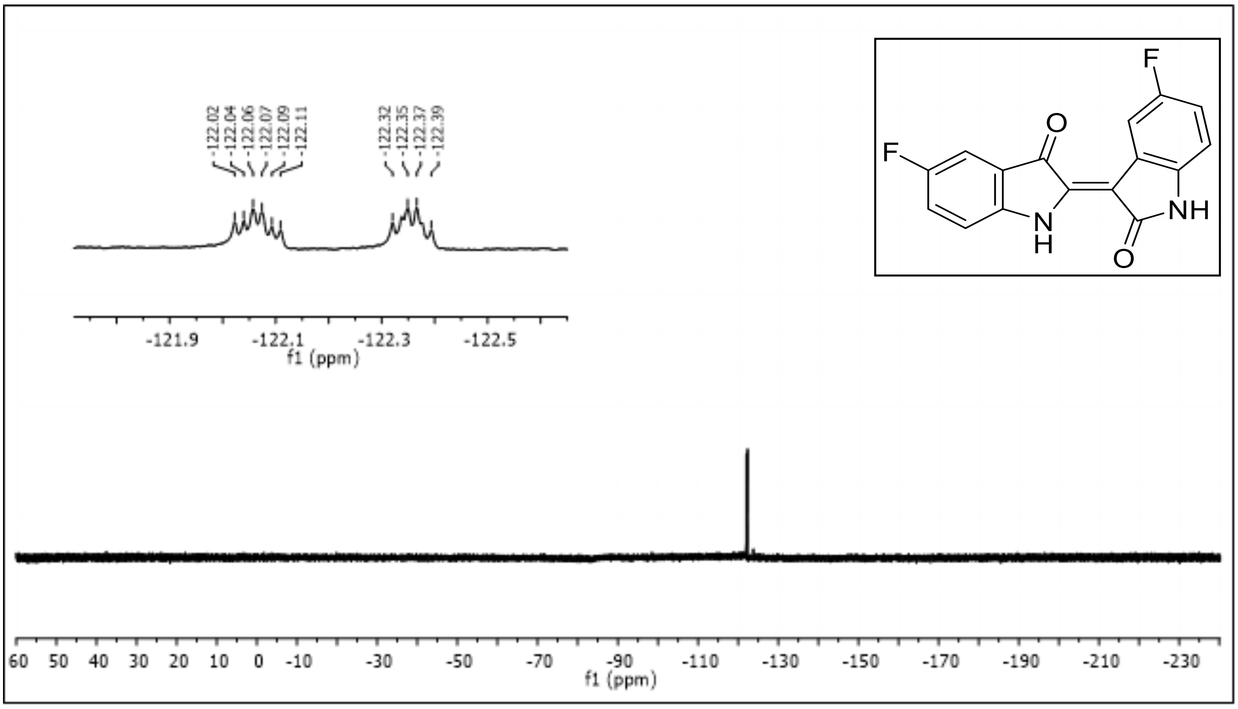

^{1}H NMR, 600 MHz, DMSO-d_6 (**2c**)

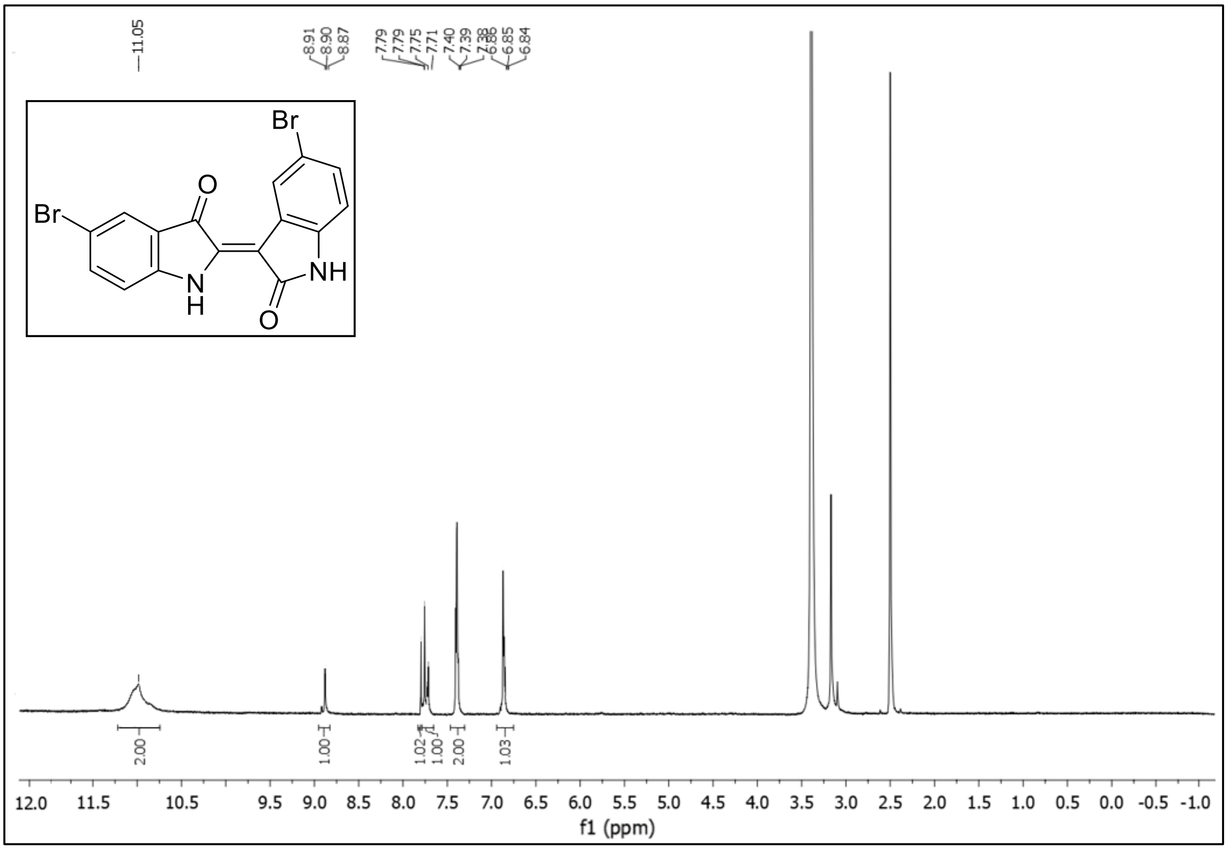

^{13}C NMR, 150 MHz, DMSO-d_6 (**2c**)

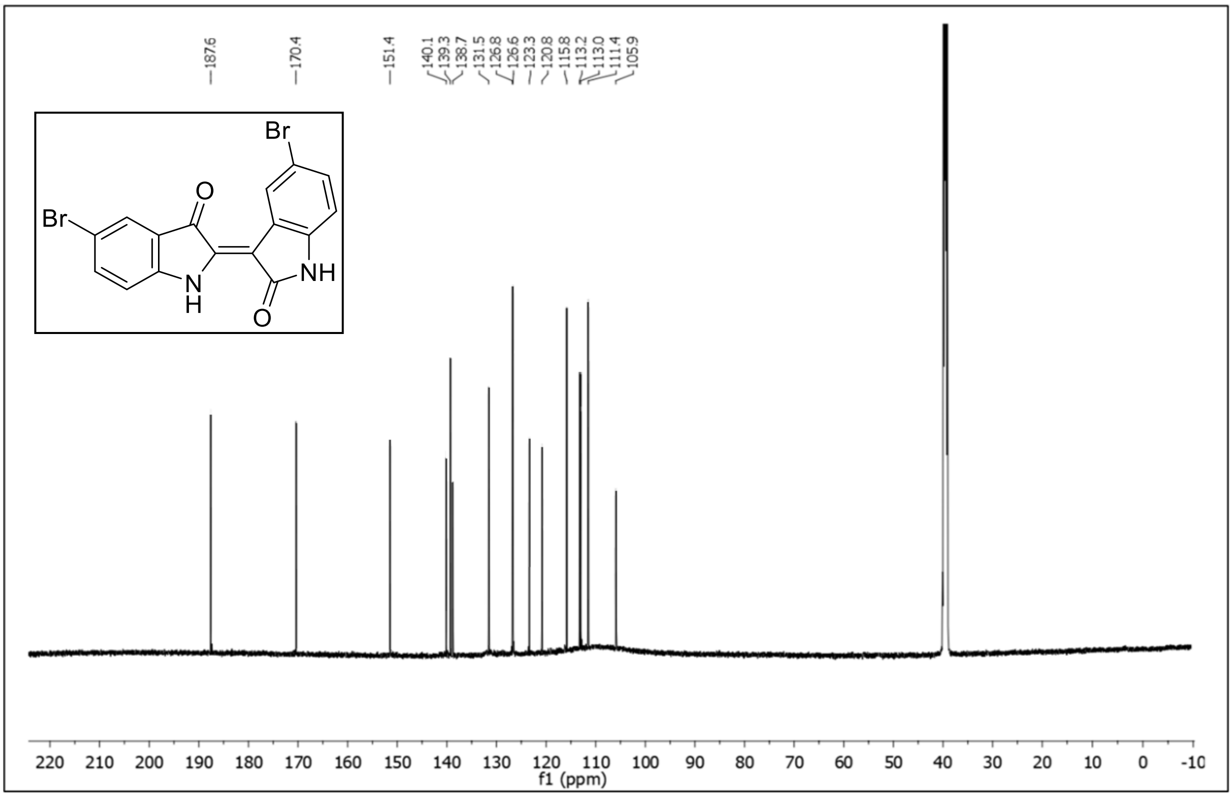

^{1}H NMR, 600 MHz, DMSO-d_6 (**2d**)

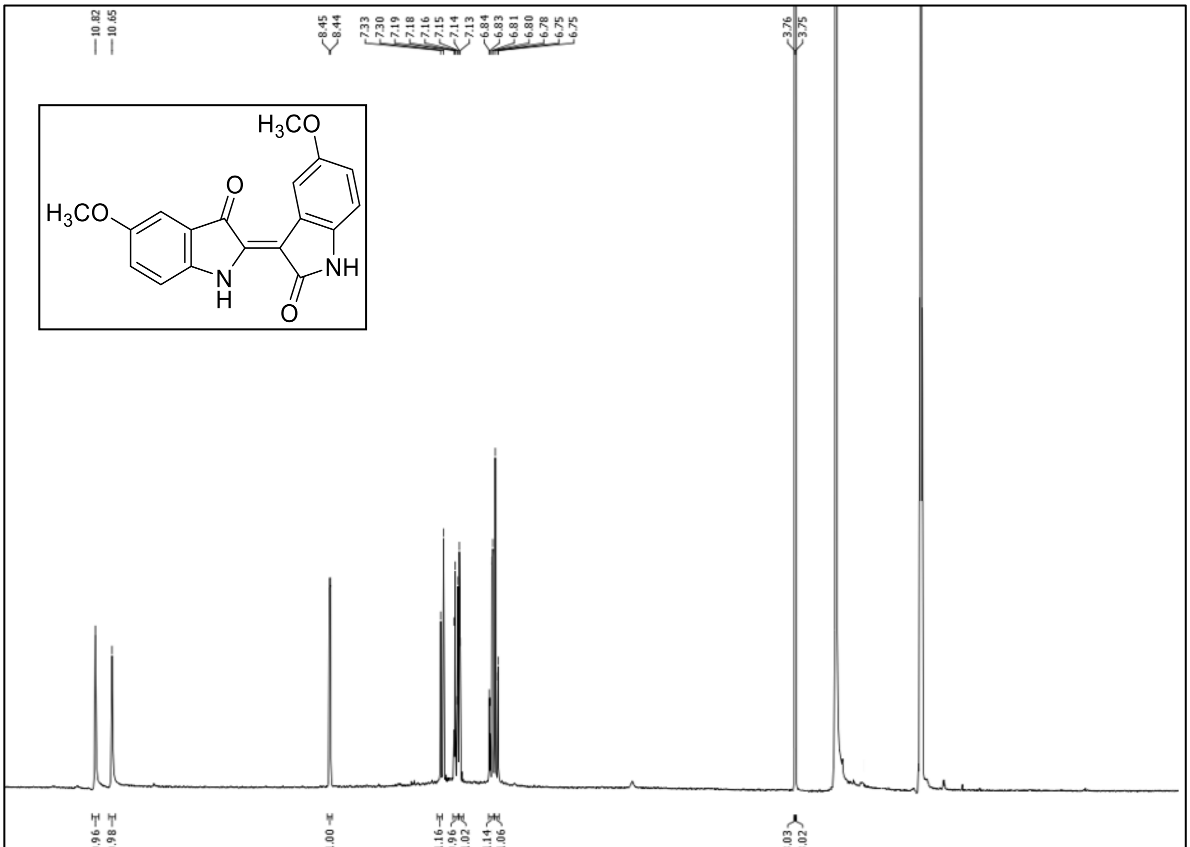

^{13}C NMR, 150 MHz, DMSO-d_6 (**2d**)

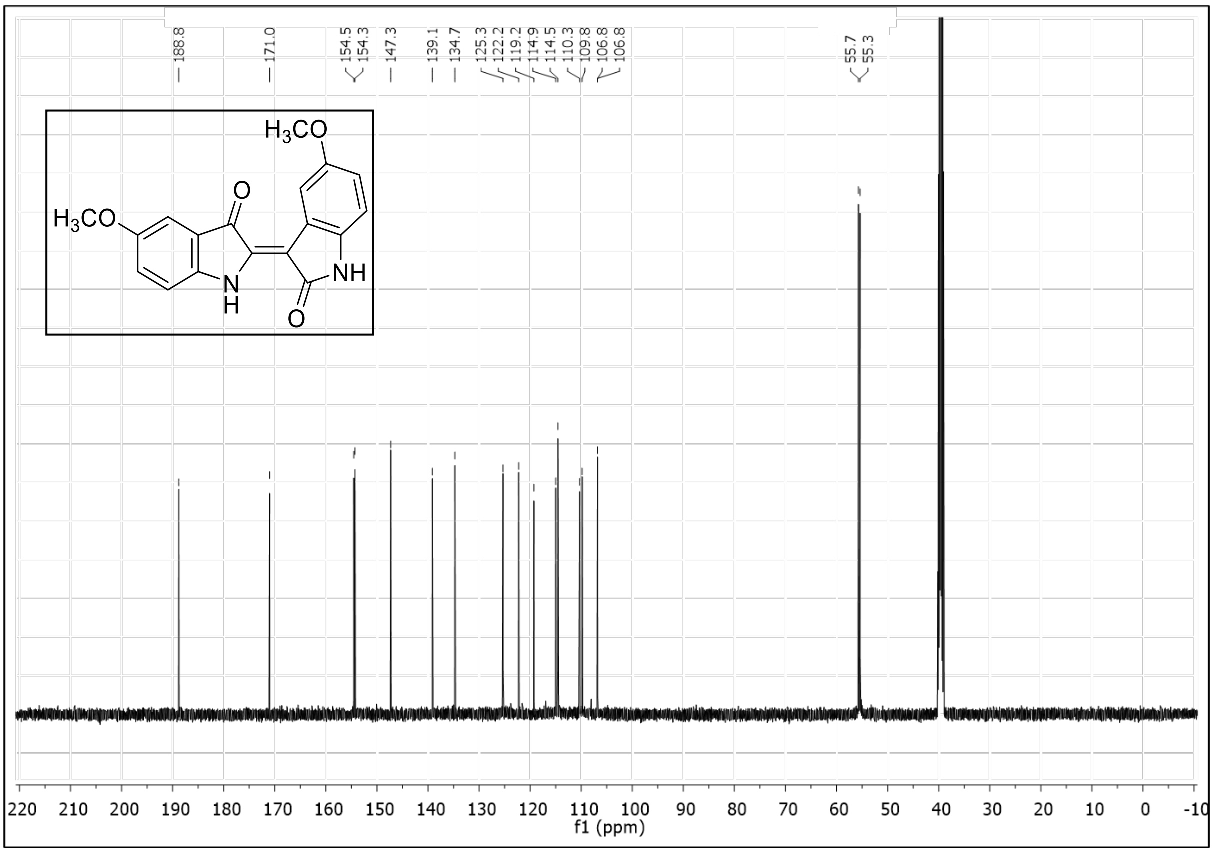

^{1}H NMR, 600 MHz, DMSO-d_6 (**2e**)

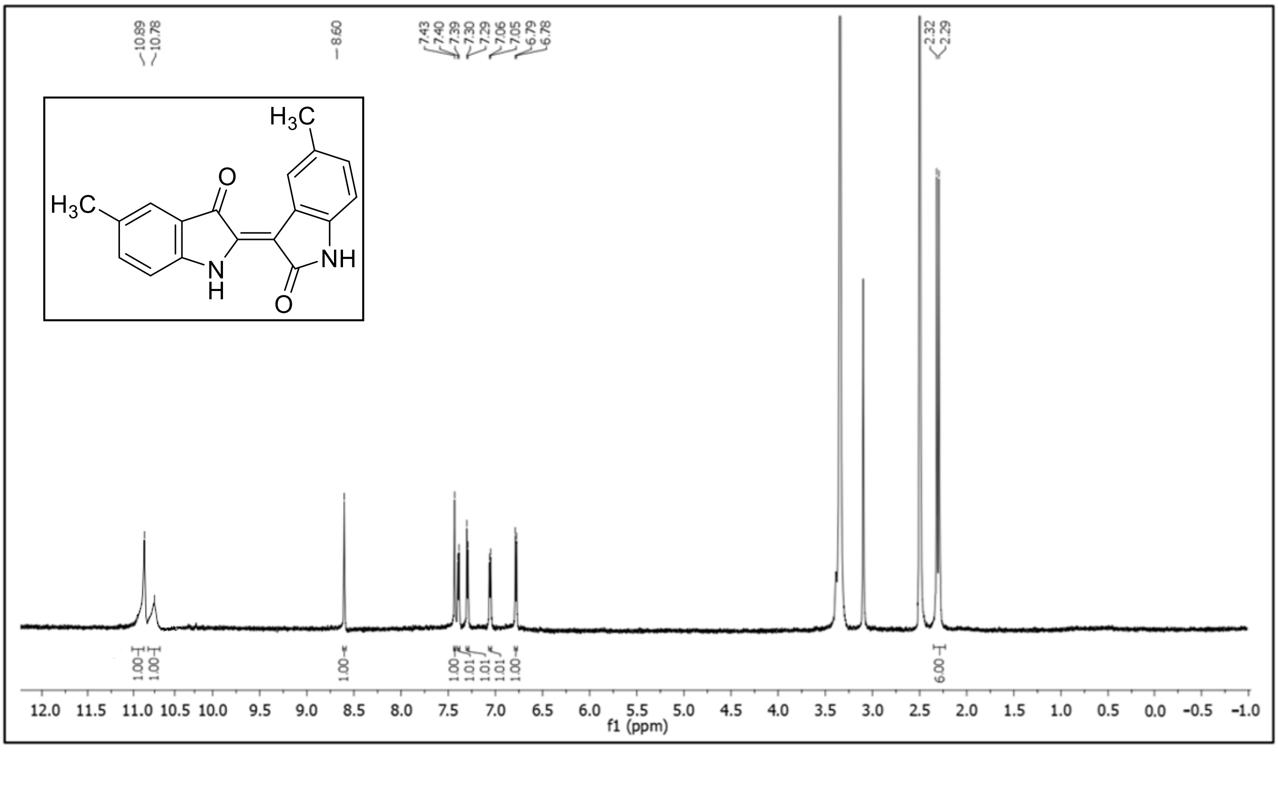

^{13}C NMR, 150 MHz, DMSO-d_6 (**2e**)

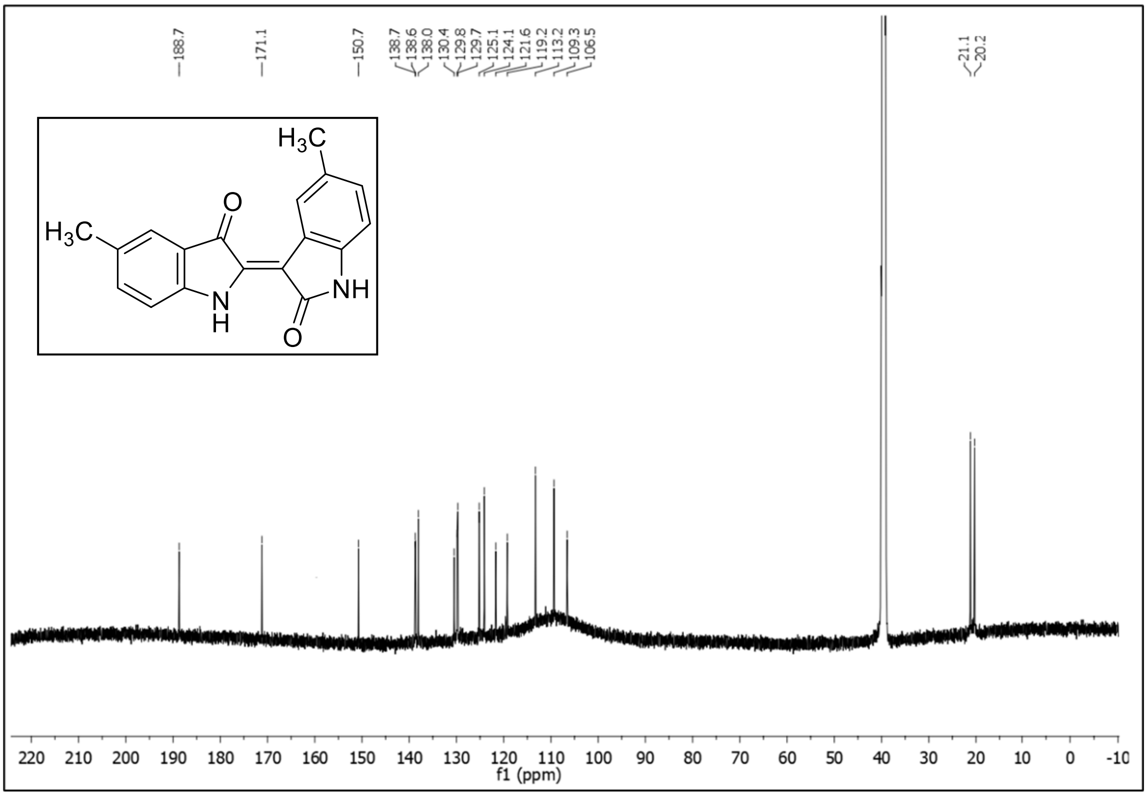

^{1}H NMR, 600 MHz, DMSO-d_6 (**2f**)

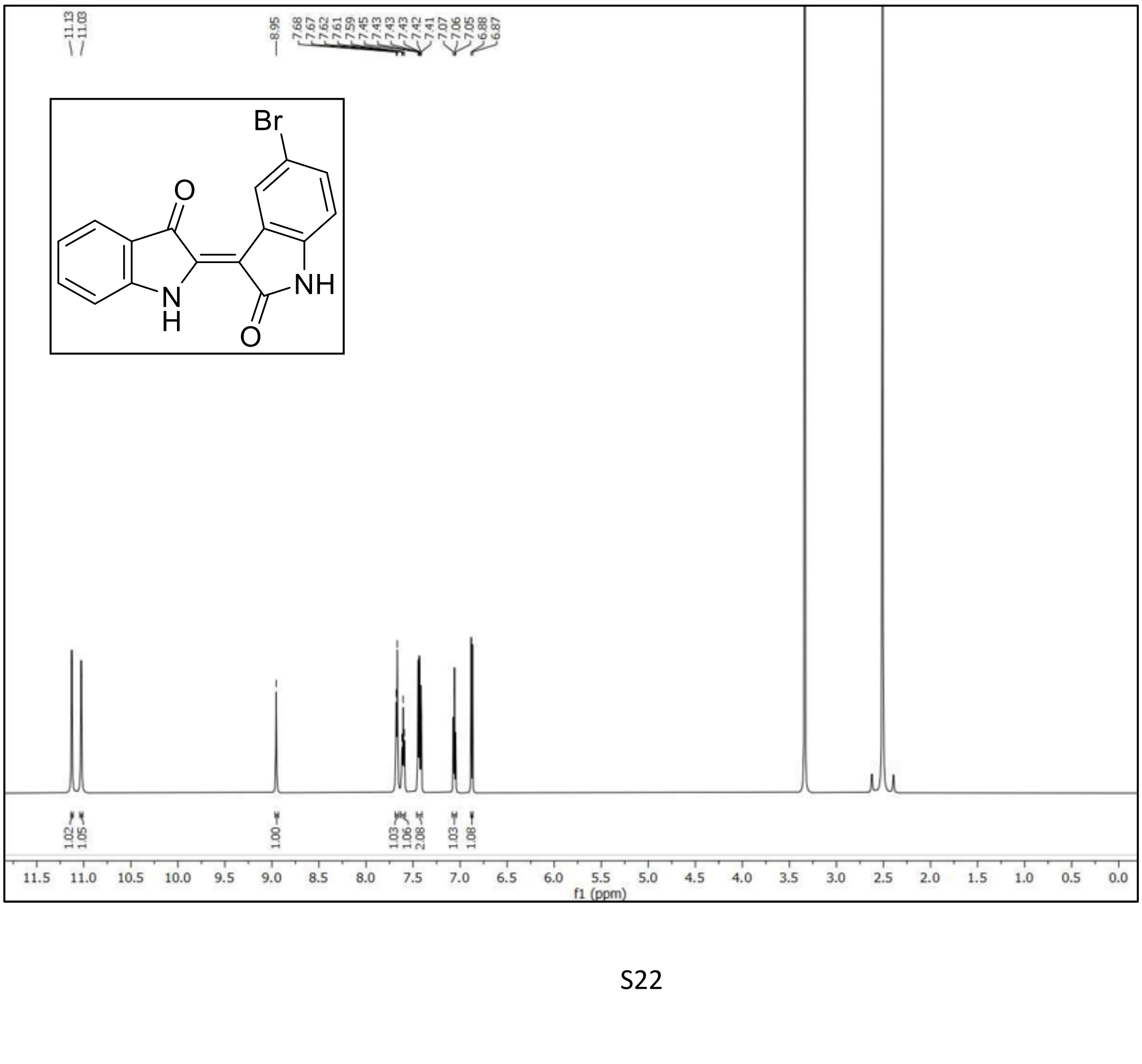

^{13}C NMR, 150 MHz, DMSO-d_6 (**2f**)

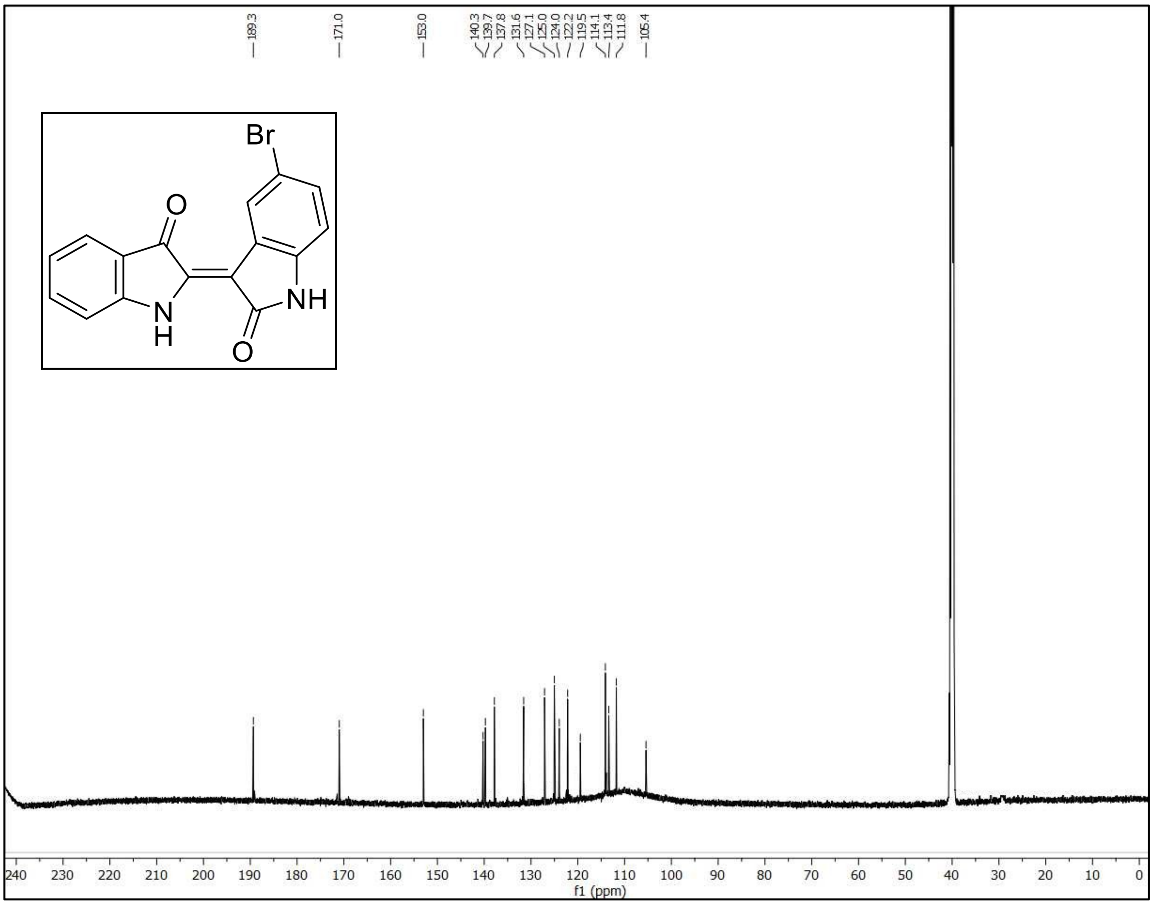

^{1}H NMR, 600 MHz, DMSO-d_6 (**2g**)

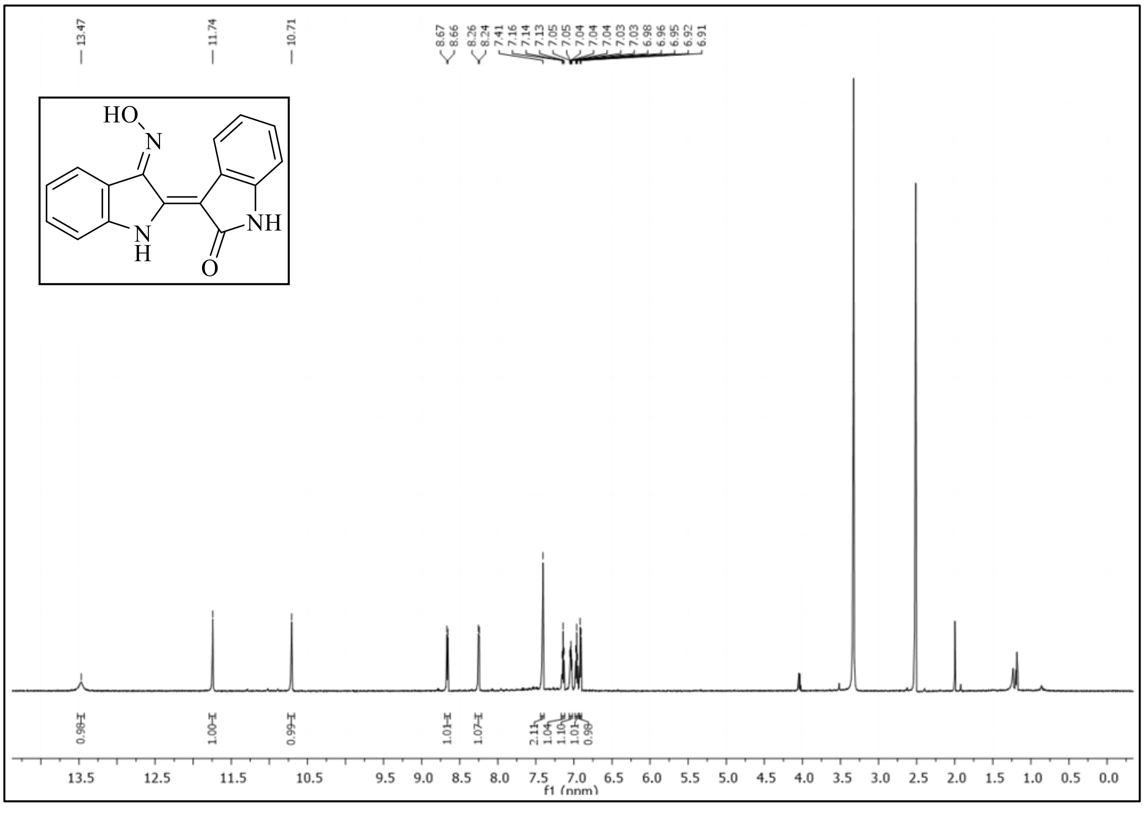

^{13}C NMR, 150 MHz, DMSO-d_6 (**2g**)

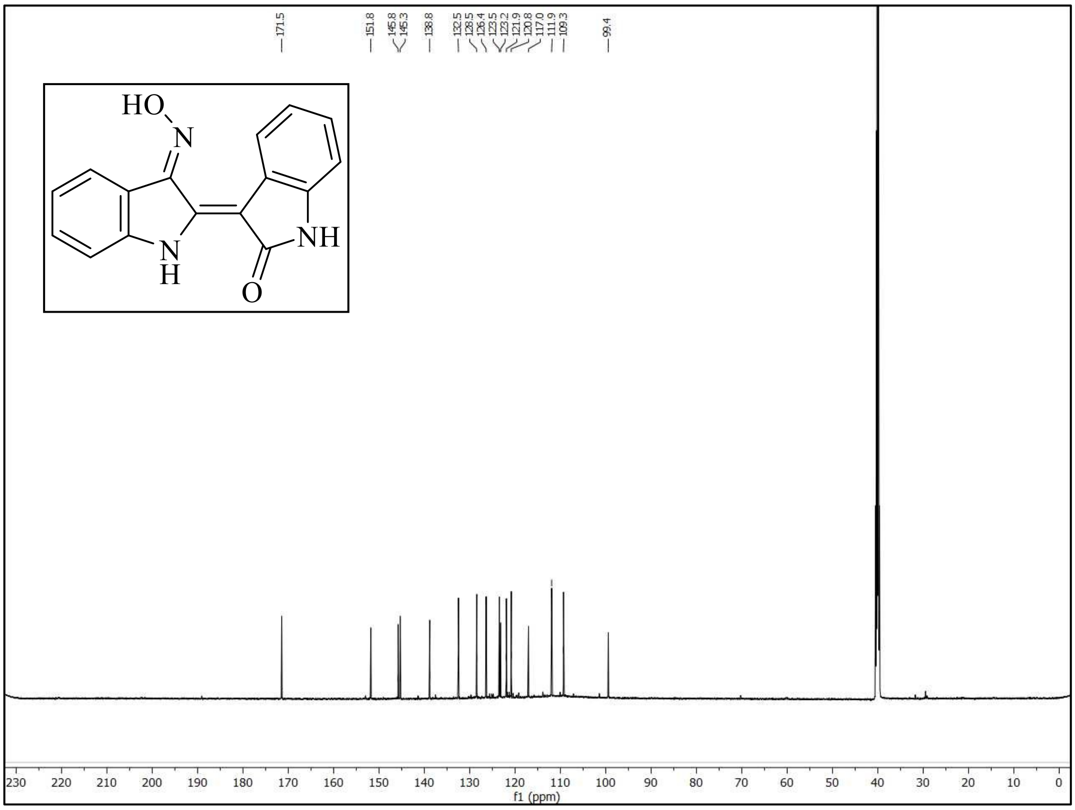

^{1}H NMR, 600 MHz, $CDCl_3$ (**3a**)

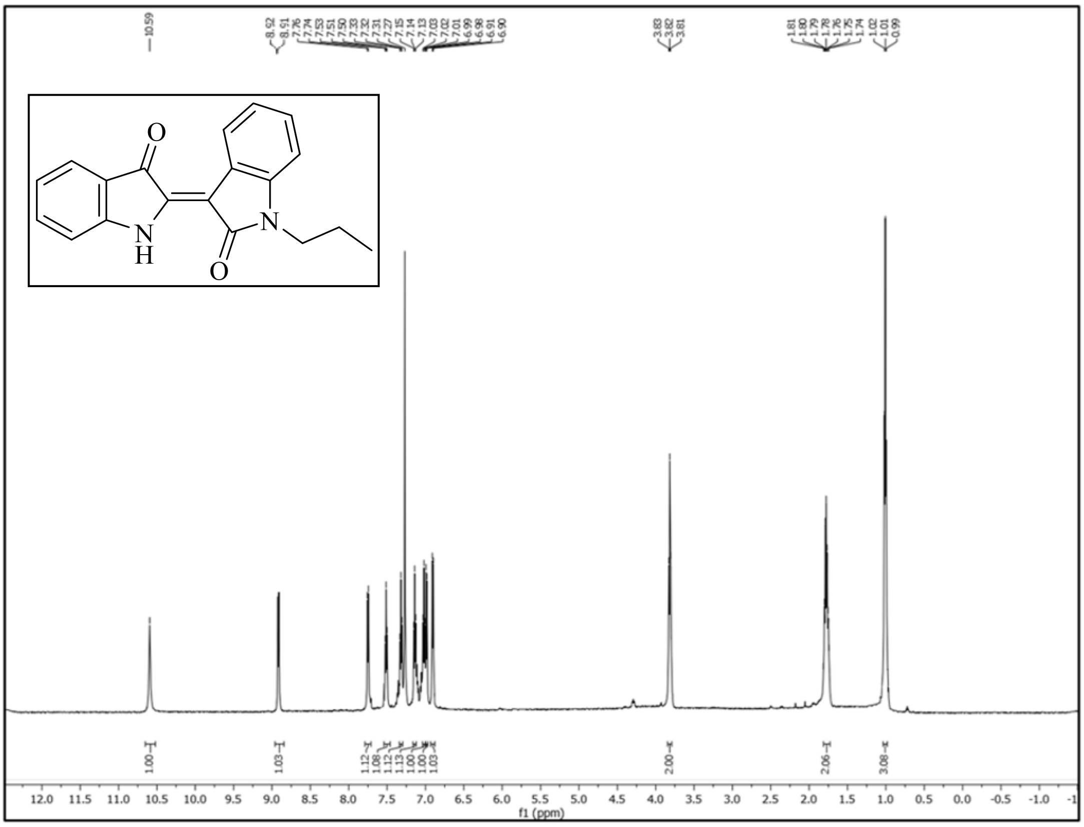

^{13}C NMR, 150 MHz, $CDCl_3$ (**3a**)

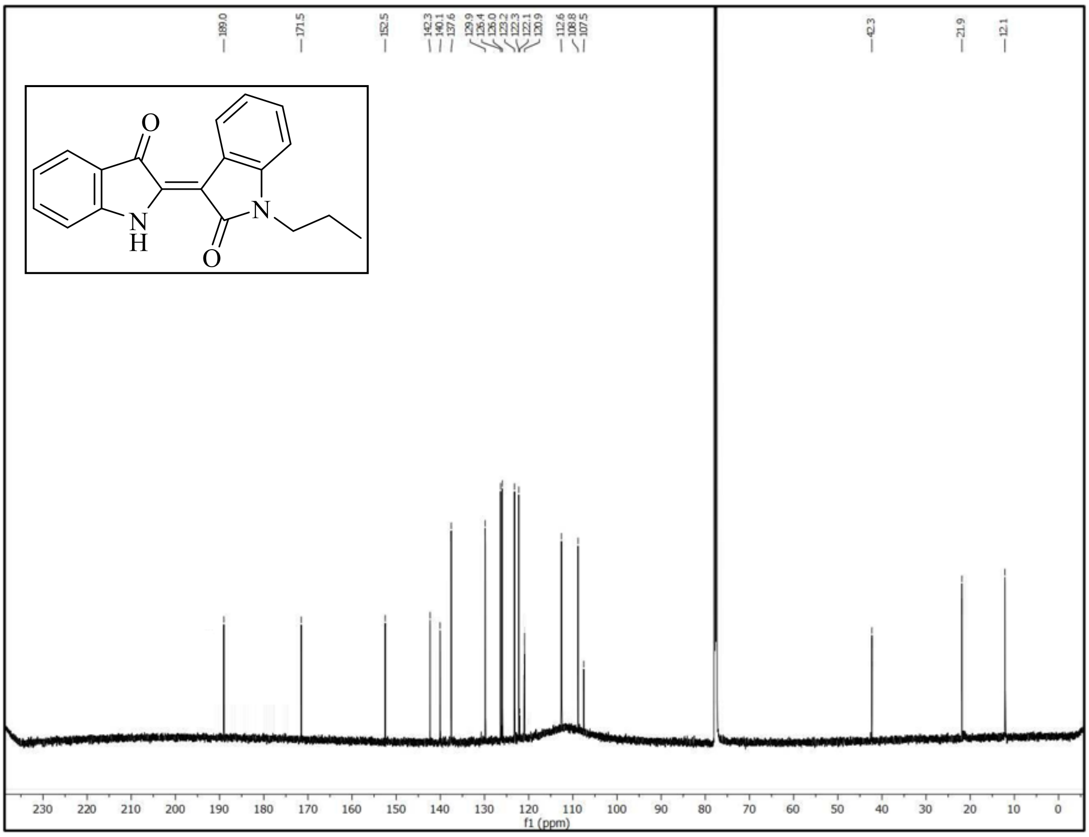

^{1}H NMR, 600 MHz, $CDCl_3$ (**4b**)

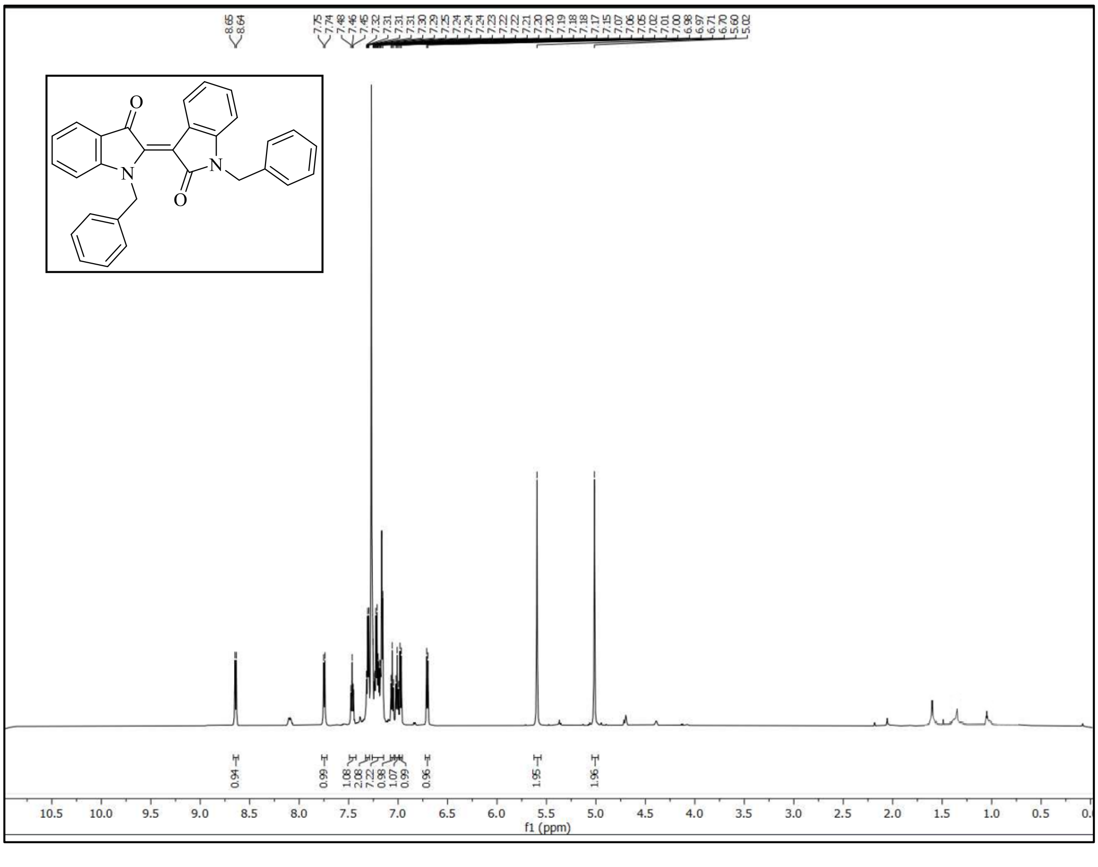

^{13}C NMR, 150 MHz, $CDCl_3$ (**4b**)

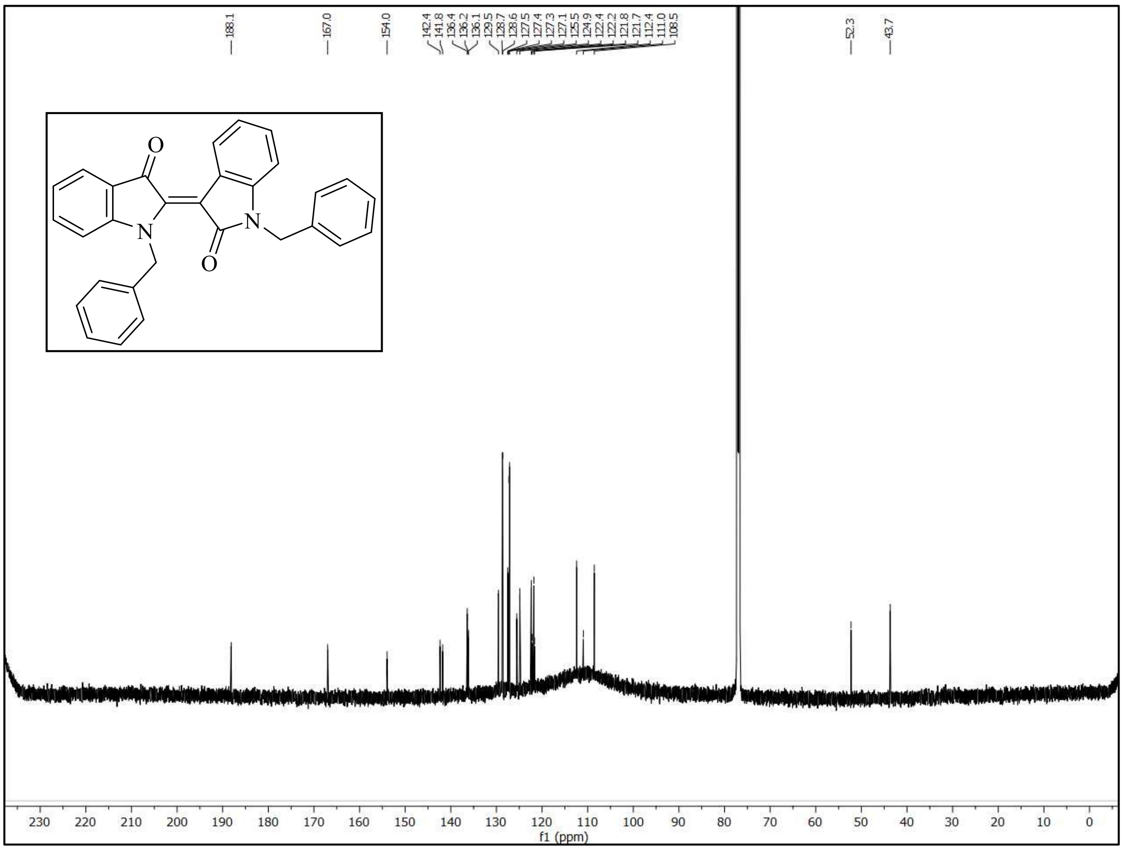

Supplement: Supplementary file 1 — Supplementary Material [file CSSC-19-e202502114-s001.pdf]
